# Timing of Hepatectomy for Resectable Synchronous Colorectal Liver Metastases: For Whom Simultaneous Resection Is More Suitable - A Meta-Analysis

**DOI:** 10.1371/journal.pone.0104348

**Published:** 2014-08-05

**Authors:** Qingyang Feng, Ye Wei, Dexiang Zhu, Lechi Ye, Qi Lin, Wenxiang Li, Xinyu Qin, Minzhi Lyu, Jianmin Xu

**Affiliations:** 1 Department of General Surgery, Zhongshan Hospital, Fudan University, Shanghai, China; 2 Department of Biostatistics, Shanghai Medical College, Fudan University, Shanghai, China; University of Pittsburgh School of Medicine, United States of America

## Abstract

**Background:**

The optimal timing of resection for synchronous colorectal liver metastases is still controversial. Retrospective cohort studies always had baseline imbalances in comparing simultaneous resection with staged strategy. Significantly more patients with mild conditions received simultaneous resections. Previous published meta-analyses based on these studies did not correct these biases, resulting in low reliability. Our meta-analysis was conducted to compensate for this deficiency and find candidates for each surgical strategy.

**Methods:**

A systemic search for major databases and relevant journals from January 2000 to April 2013 was performed. The primary outcomes were postoperative mortality, morbidity, overall survival and disease-free survival. Other outcomes such as number of patients need blood transfusion and length of hospital stay were also assessed. Baseline analyses were conducted to find and correct potential confounding factors.

**Results:**

22 studies with a total of 4494 patients were finally included. After correction of baseline imbalance, simultaneous and staged resections were similar in postoperative mortality (RR = 1.14, P = 0.52), morbidity (RR = 1.02, P = 0.85), overall survival (HR = 0.96, P = 0.50) and disease-free survival (HR = 0.97, P = 0.87). Only in pulmonary complications, simultaneous resection took a significant advantage (RR = 0.23, P = 0.003). The number of liver metastases was the major factor interfering with selecting surgical strategies. With >3 metastases, simultaneous and staged strategies were almost the same in morbidity (49.4% vs. 50.9%). With ≤3 metastases, staged resection caused lower morbidity (13.8% vs. 17.2%), not statistically significant.

**Conclusions:**

The number of liver metastases was the major confounding factor for postoperative morbidity, especially in staged resections. Without baseline imbalances, simultaneous took no statistical significant advantage in safety and efficacy. Considering the inherent limitations of this meta-analysis, the results should be interpret and applied prudently.

## Introduction

Colorectal cancer (CRC) remains one of the most common malignancies all over the world [1], [2]. Up to 50% of patients with CRC might have liver metastases during the course of the disease [3], and 15% to 20% have synchronous colorectal liver metastases (SCRLM) at the time of diagnosis [4], [5]. Liver resection has been considered the only treatment offering the chance for a cure and long-term survival of SCRLM. However, optimal timing of liver surgery for upfront resectable synchronous metastases remains controversial. Traditionally, most investigators have recommended a staged strategy with resection of primary colorectal tumor followed by chemotherapy, then hepatectomy 2 to 3 months later. But over the last 20 years, simultaneous resection of upfront resectable SCRLM has been widely carried out due to advances in oncological concepts and surgical techniques.

The safety and efficacy of simultaneous resection has been demonstrated by some recent studies [6]–[9]. However, the consensus has not been reached. In traditional opinions, simultaneous resection would result in greater surgical trauma, and surgeons always selected simultaneous resection for patients with mild conditions. For this reason, there were significant baseline imbalances between simultaneous and staged resection groups. The conclusion of previous meta-analyses could have low reliability without correction of the imbalances [10]–[12]. In addition, the selection of surgical strategy is only one of potential factors affecting the prognosis of patients with SCRLM. Other confounding factors could interfere with the surgical strategy. Patients with different clinical characteristics might be suitable for different strategy. We therefore conducted this meta-analysis to evaluate the safety and efficacy of simultaneous resection strategy with correction of baseline imbalance, and tried to find candidates for each surgical strategy.

## Materials and Methods

The methods of literature search, inclusion and exclusion criteria, outcome measures, and methods of statistical analysis were defined in a protocol according to the PRISMA (Preferred Reporting Items for Systematic Reviews and Meta-Analyses) checklist and flow diagram. Considering the large number of retrospective cohort studies previously reported, the Meta-analysis of Observational Studies in Epidemiology (MOOSE) recommendations for study reporting [13] was also followed. To ensure the scientificity of analysis procedures, we also gained support from the Cochrane group of Zhongshan Hospital, Fudan University and the Department of Biostatistics, Shanghai Medical College, Fudan University.

### Literature search

Literature search was performed to identify all relevant studies that compared the outcomes following simultaneous resection or staged resection for the treatment of SCRLM. The databases including PubMed, Web of Science, Embase and Cochrane Central Register of Controlled Trials were searched systematically for all articles published from January 2000 to April 2013. Database-specific search terms of simultaneous resection and staged resection were combined with truncated search terms using the wildcard (“*”) character to ensure the integrity of search results. Additionally, the “related articles” function and manual searches for reference lists were used to broaden the search. When the results of a single study were reported in more than one publication, only the most complete and latest data were included.

### Selection of studies

All clinical studies in which simultaneous resection was compared with staged resection in SCRLM were selected. In simultaneous resection, primary colorectal tumor and liver metastases were resected in one operation. In staged resection, primary tumor was resected first, then a second hepatectomy was conducted during the following 2 to 3 months. The inclusion criteria were as follows: (1) clinical trials or cohort studies; (2) studies with a definition of SCRLM as diagnosed liver metastases before or during surgery; (3) primary colorectal tumors and liver metastases were both resectable at diagnosis; (4) studies reported at least one primary outcome; (5) studies published or accepted for publication as full-length articles.

The following studies were excluded: (1) studies lacking a control group or in which the control group was unreasonable; (2) “Liver First” resection which meant a hepatectomy first, followed by a second primary tumor resection; (3) studies from which it was impossible to extract or calculate the data of interest; (4) low-quality studies.

Two reviewers independently screened the literature and determined whether to include each study by reading the title, abstract, and full text. Disagreement between the two reviewers was resolved by discussion or third-party arbitration, if necessary.

### Quality assessment

The quality of clinical trials was evaluated using the seven-point Jadad ranking system [14], and the quality of cohort studies was evaluated using the Newcastle-Ottawa Scale (NOS) [15]. All evaluations were independently conducted by two reviewers. Disagreement between the two reviewers was resolved by discussion or third-party arbitration, if necessary. Low quality was defined as follows: a score <2 on the Jadad Scale or a score <6 on the NOS.

### Data extraction and measurement

Outcomes assessed were primary parameters of postoperative mortality, morbidity, overall survival and disease-free survival. Secondary outcomes were number of patients who need blood transfusion during the surgery, and length of hospital stay. Studies included in the meta-analysis should report at least one primary outcome. Postoperative mortality was defined as death during postoperative hospitalization or within 30 days after hepatectomy. Postoperative morbidity included complications directly related to primary colorectal cancer resection, to hepatectomy, and others during postoperative hospitalization or within 30 days after surgery. Overall survival (OS) and disease-free survival (DFS) were calculated since the hepatectomy was performed, and patients died within 90 days were excluded. Data of long-term survival was extracted from the Kaplan-Meier curves as described by Tierney et al. [16]. In staged resection groups, patients need blood transfusion and hospital stay were calculated as a sum of the primary tumor resection and the following hapetectomy. Minor hepatectomy was defined as resection of <3 liver segments, and major hepatectomy was defined as resection of ≥3 liver segments. In terms of primary tumor location, the transverse colon was included in the right-sided, and the sigmoid colon was included in the left-sided.

A data form was designed for extraction, consisting of four parts: patient characteristics, surgery-related factors, study outcomes, and analysis methods. The extracted data were independently checked by two reviewers. Emails were sent to each author for detailed data unavailable from the published article.

### Statistical methods

The meta-analysis was performed using RevMan software ver. 5.0.23 and SPSS software ver. 19. The reported risk ratio (RR) and weighted mean difference (WMD) with 95% confidence interval (CI) were used to assess the short-term outcomes, and the odds ratio (OR) was used to assess the baseline imbalance in the analysis. Continuous variables reported as medians were converted to means using the technique described by Hozo et al [17]. For long-term outcomes, the hazard ratio (HR) was used to pool the survival curves as described by Tierney et al [16]. The statistical tests were two-sided, and P<0.05 was considered statistically significant.

Statistical heterogeneity among trials was assessed with Cochrane's Q statistic, and was considered statistically significant when the Cochrane Q test P value was ≤0.1. In addition, a transformation of Q test, the I^2^ statistic (I^2^ = 100%×(Q−df)/Q), was used to assess the consistency of the effect sizes. The I^2^ value of less than 25% was defined to represent low heterogeneity, a value between 25 and 50% was defined as moderate heterogeneity, and a value of >50% was defined as high heterogeneity [18]. A fixed-effects model was used when no significant heterogeneity was detected. If heterogeneity existed (Cochrane Q test P value >0.1, or I^2^>50%), a random-effects model was used for the meta-analysis instead.

Sensitivity analyses were performed by consecutively omitting every study from the meta-analysis (leave-one-out procedure) to determine the extent to which the combined risk estimate might be affected by individual studies. Funnel plots were used to screen for publication bias.

## Results

### Studies and patients

**Figure 1:**
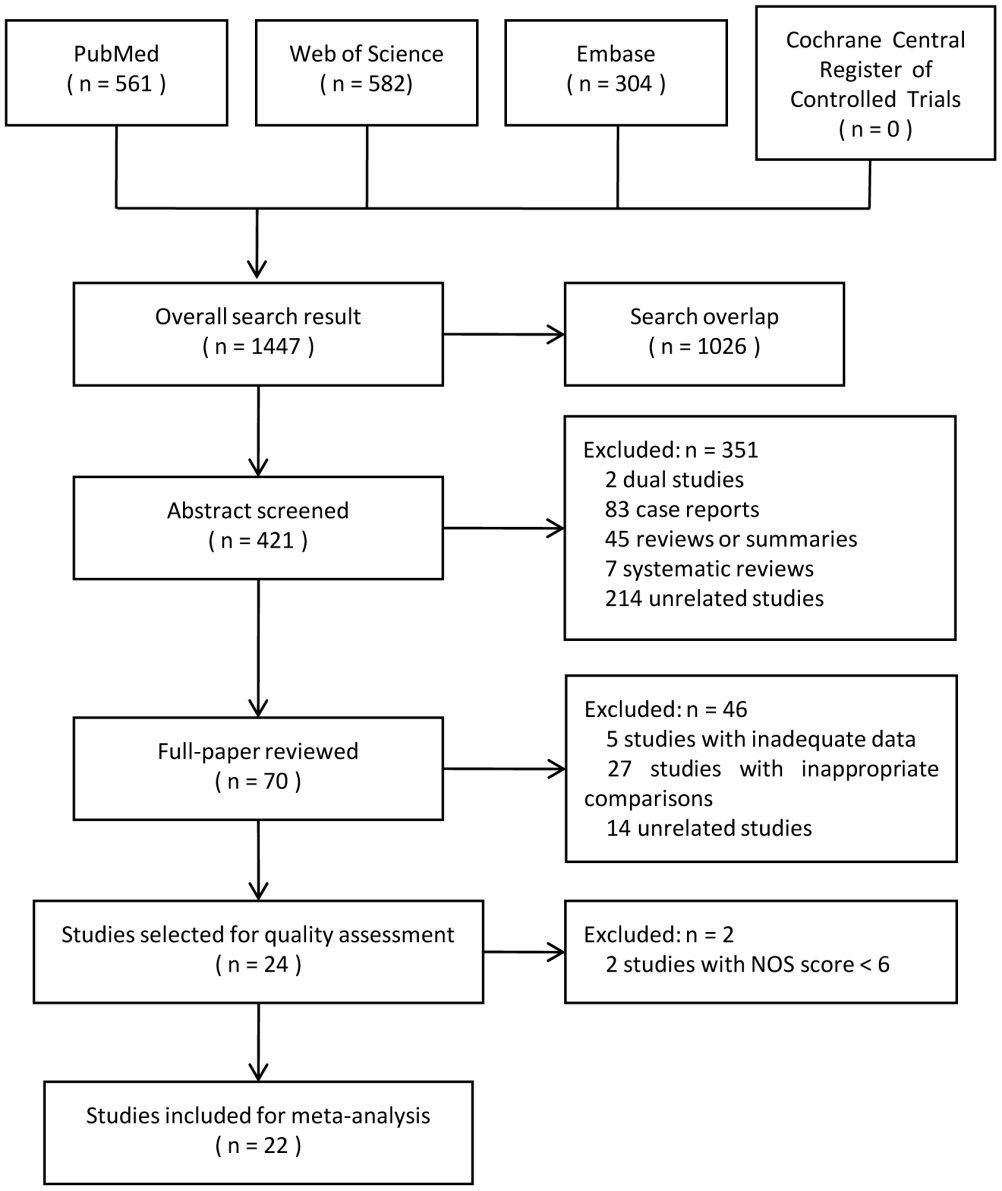
Study search process.

After searching the databases, 24 studies were selected for quality assessment. The search process is shown in Figure 1, and the search strategy is shown in Information S1. All these studies were retrospective cohort studies, and one of them was case-matched [19]. No randomized controlled trial was found. NOS was used for quality assessment, and only studies with high quality (NOS score ≥6) were included for the following meta-analysis.

After excluding 2 studies of low quality (Vigano et al. [20] and van der Pool et al. [21]), 22 studies were finally included, with the scores ranged from 6 to 9. All the 22 studies got full scores in “Cohort Selection”. The quality defects mainly existed in “Cohort Comparability”: only 10 studies analyzed the potential confounding factors when comparing the outcomes. In addition, some studies didn't have long-enough follow-up time, nor explained the missing cases. The quality assessment is detailed in Table S1.

A total of 4494 patients from 22 studies were finally included in the meta-analysis, of which 1708 (38.0%) underwent simultaneous resection, and 2786 (62.0%) underwent staged resection. The detailed characteristics of the studies are listed in Table 1.

**Table 1:** Characteristics of studies included for Meta-analysis.

				Simultaneous resection			Staged resection			
Authors	Year	Country	Total patients	Patients	Age (mean)	Male/Female	Patients	Age (mean)	Male/Female	Quality score (NOS)
Abbott et al. [28]	2012	USA	144	60	57.5*	40/20	84	53.3*	49/35	9
Alexandrescu et al. [6]	2012	Romania	142	117	59.0	53/64	25	56.7	9/16	7
Brouquet et al. [7]	2010	USA	115	43	56.0*	23/20	72	54.5*	44/28	8
Capussotti et al. [29]	2007	Italy	127	70	64.9	40/30	57	60.8	35/22	8
Chua et al. [30]	2004	USA	96	64	63.0	39/25	32	61.0	18/14	6
de Haas et al. [26]	2010	France	228	55	56.0	28/27	173	58.0	107/66	9
Hu et al. [31]	2013	China	53	40	57.2	25/15	13	52.4	6/7	8
Luo et al. [32]	2010	China	405	129	58.0	76/53	276	60.0	156/120	6
Martin et al. [33]	2003	USA	240	134	60.0*	69/65	106	56.8*	61/45	7
Martin et al. [34]	2009	USA	230	70	55.3*	38/32	160	57.5*	91/69	7
Mayo et al. [9]	2013	USA	976	329	60.0	185/144	647	58.0	396/251	8
Moug et al. [19]	2010	UK	64	32	69.0*	18/14	32	67.0*	21/11	7
Reddy et al. [35]	2007	USA	610	135	57.0*	84/51	475	58.0*	277/198	6
Slupski et al. [8]	2009	Poland	89	28	59.4	18/10	61	60.2	34/27	6
Tanaka et al. [22]	2004	Japan	76	39	65.0	20/19	37	64.0	25/12	7
Thelen et al. [23]	2007	German	219	40	60.5	24/16	179	59.7	96/84	8
Turrini et al. [36]	2007	France	119	57	60.0	26/31	62	59.0	28/34	8
Vassiliou et al. [37]	2007	Greece	103	25	63.0	15/10	78	61.0	47/31	6
Wang et al. [38]	2008	China	83	37	57.0*	22/15	46	55.0*	31/15	8
Weber et al. [39]	2003	France	97	35	58.0	18/17	62	60.0	31/31	7
Xu et al. [40]	2009	China	175	96	48.2	54/42	79	52.3	49/30	7
Yan et al. [41]	2007	Australia	103	73	60.0	33/40	30	59.0	15/15	6

* Means were converted from medians. NOS: Newcastle-Ottawa Scale.

### Short-term outcomes

**Figure 2:**
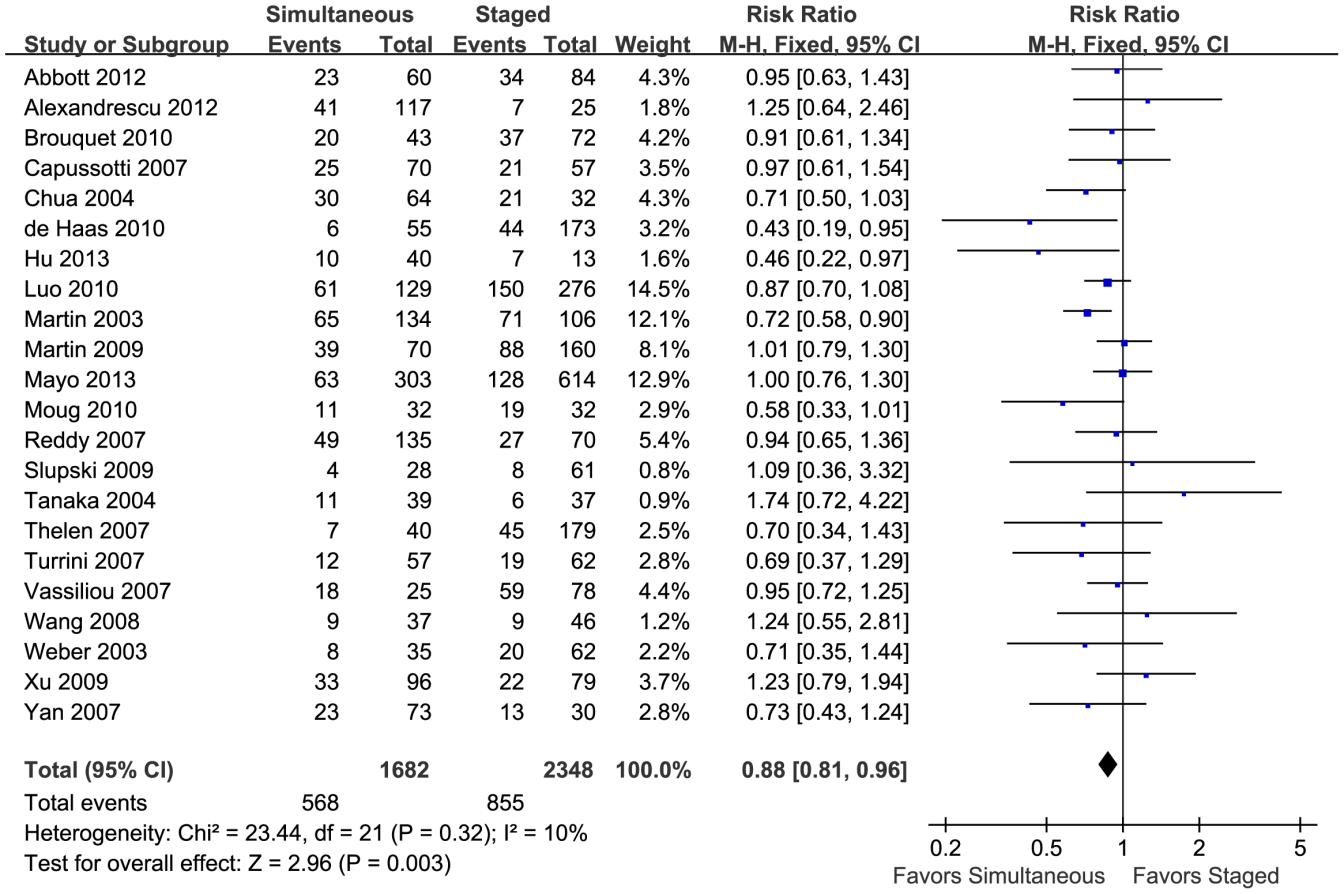
Pooled postoperative morbidity. Forest plots displayed the results of the meta-analysis comparing postoperative complication morbidity following simultaneous resection vs. staged resection for SCRLMs. M-H: Mantel-Haenszel method. Fixed: The heterogeneity test showed no significant heterogeneity, and fixed effect model was used. CI: confidence interval. Favours Simultaneous: With results on this side, simultaneous group had lower postoperative mortality. Favours Staged: With results on this side, staged group had lower postoperative mortality.

To evaluate the safety of simultaneous and staged resection for treating SCRLM, RRs of postoperative mortality and morbidity were calculated using the data extracted from the 22 included studies. For postoperative mortality, the pooled results showed no significant difference between simultaneous and staged resection (RR = 1.14, 95%CI = [0.77, 1.69], P = 0.52, details in Figure S1). However, simultaneous resection showed a significant advantage in reducing the postoperative morbidity (RR = 0.88, 95%CI = [0.81, 0.96], P = 0.003, details in Figure 2), which meant that simultaneous resections were safer for patients with SCRLM.

The analyses of different complications showed that the advantage of simultaneous resections mainly came from the lower morbidity of cardiac complications (RR = 0.43, 95%CI = [0.22, 0.84], P = 0.01) and pulmonary complications (RR = 0.58, 95%CI = [0.40, 0.83], P = 0.003). There were no significant differences between the two groups in wound infection (RR = 0.77, 95%CI = [0.55, 1.08], P = 0.13), anastomotic leakage (RR = 1.23, 95%CI = [0.58, 2.59], P = 0.59), bile leakage (RR = 0.66, 95%CI = [0.39, 1.11], P = 0.12), subphrenic or perihepatic abscess (RR = 1.33, 95%CI = [0.87, 2.02] P = 0.19), or hepatic insufficiency and failure (RR = 0.80, 95%CI = [0.46, 1.37], P = 0.41). Detailed results are shown in Figure S2.

### Long-term outcomes

**Figure 3:**
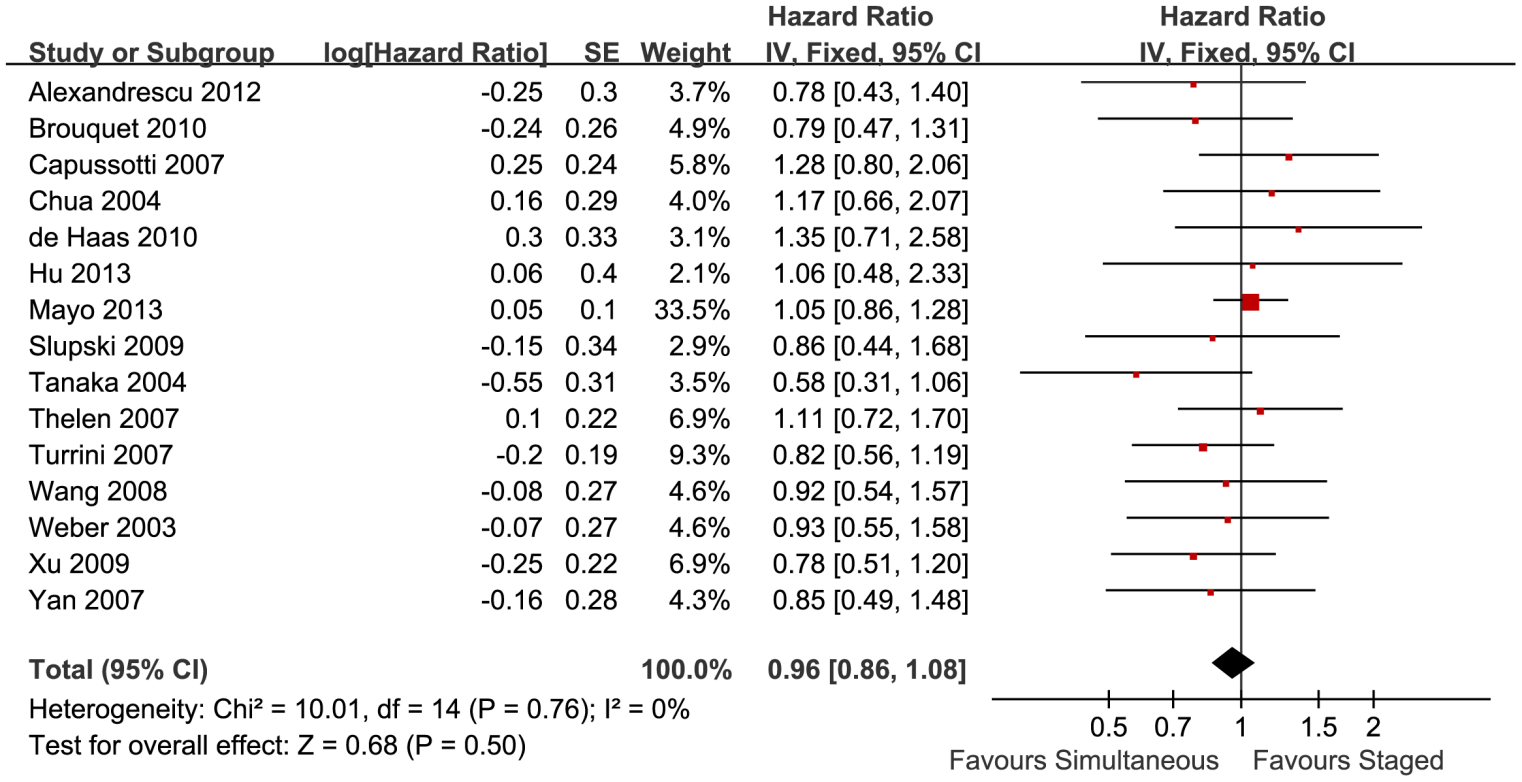
Subtype analyses of pooled postoperative morbidity. Forest plots displayed the results of the meta-analysis comparing overall survival following simultaneous resection vs. staged resection for SCRLMs. IV: Inverse Variance method. Fixed: The heterogeneity test showed no significant heterogeneity, and fixed effect model was used. CI: confidence interval. Favours Simultaneous: With results on this side, simultaneous group had longer overall survival. Favours Staged: With results on this side, staged group had longer overall survival.

To evaluate the efficacy of simultaneous and staged resections for treating SCRLM, HRs of overall and disease-free survival were summarized using data extracted from Kaplan-Meier curves. Of all 22 studies, 15 studies reported the Kaplan-Meier curves of overall survival, with a total of 2639 patients. The pooled results showed no significant difference between simultaneous and staged resections (HR = 0.96, 95%CI = [0.86, 1.08], P = 0.50, details in Figure 3). Additionally, 6 studies reported the disease-free survival, with a total of 698 patients. And there was no significant difference between the two groups (HR = 0.97, 95%CI = [0.64, 1.47], P = 0.87, details in Figure S3). The pooled results showed similar long-term outcomes of both simultaneous and staged resections.

### Secondary outcomes

9 studies reported the number of patients who need blood transfusion. The pooled results showed no significant difference between simultaneous and staged resections (RR = 1.13, 95%CI = [0.97, 1.32], P = 0.12, details in Figure S4).

15 studies reported the length of hospital stay. Compared to staged resections, simultaneous resections significantly reduced about 5.53 days of hospital stay (95%CI = [−6.42, −4.64], P<0.00001, details in Figure S5).

### Baseline imbalances and subgroup analyses

**Figure 4:**
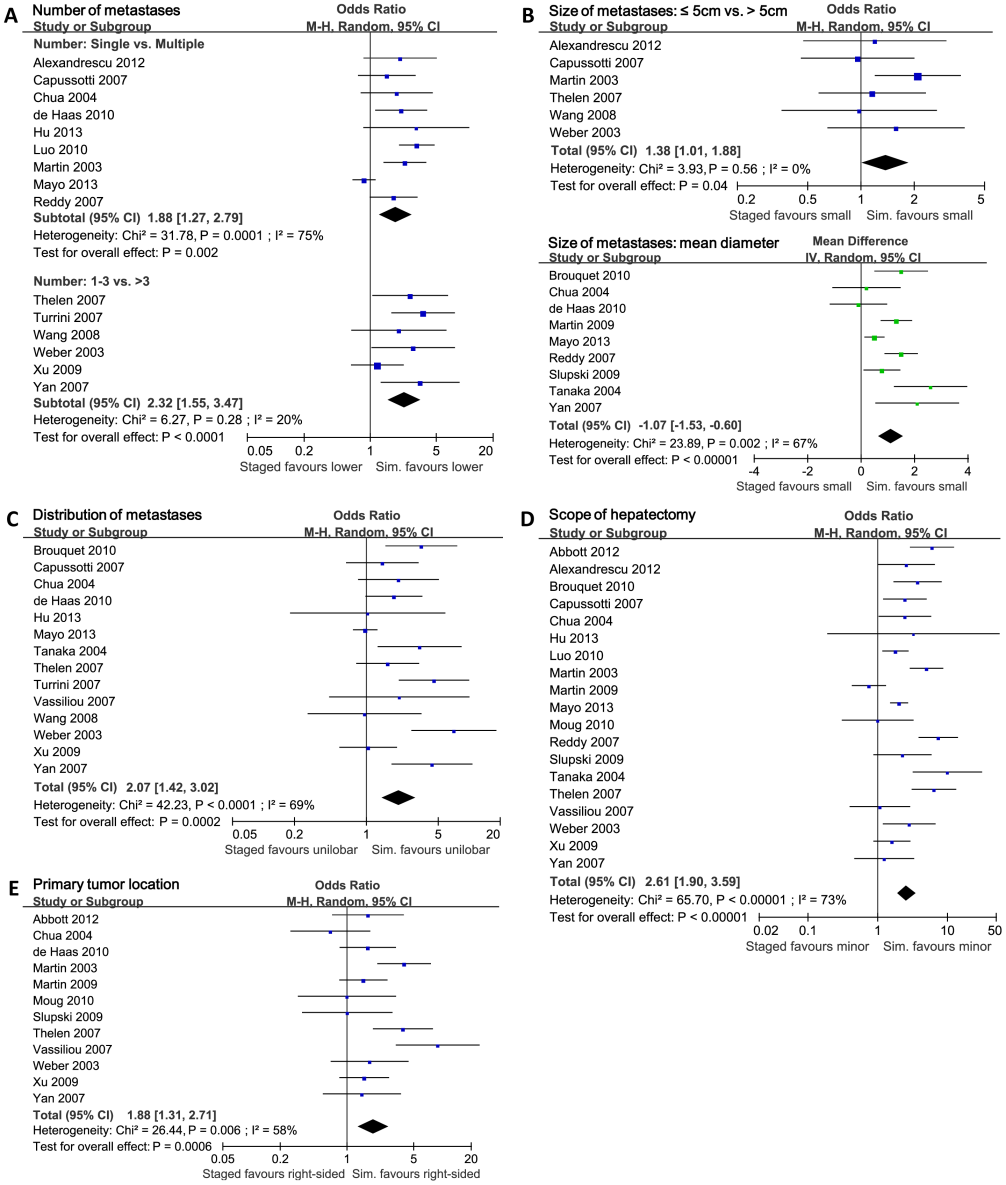
Pooled analyses of baseline imbalances. Forest plots displayed the potential confounding factors found by baseline analyses. M-H: Mantel-Haenszel method. IV: Inverse Variance method. Random: In some subgroups there were significant heterogeneity, and random effect model was used. CI: confidence interval. **A**) Baseline analysis on number of liver metastases. Staged/Sim. favours lower: more patients in staged/simultaneous group had lower number of metastases. **B**) Baseline analysis on size of liver metastases. Staged/Sim. favours small: more patients in staged/simultaneous group had smaller size of metastases. **C**) Baseline analysis on distribution of liver metastases. Staged/Sim. faours unilobar: more patients in staged/simultaneous group had unilobar liver metastases. **D**) Baseline analysis on scope of hepatectomy. Staged/Sim. favours minor: more patients in staged/simultaneous group received minor hepatectomy. **E**) Baseline analysis on primary tumor location. Staged/Sim. favours right-sided: more patients in staged/simultaneous group had primary tumor located at right-sided colon.

The pooled analysis previously mentioned showed that simultaneous resections had significant advantages in reducing postoperative complications. However, all studies included were retrospective studies, and lacked randomization process in patient enrollment. There were significant baseline imbalances between simultaneous and staged groups in several studies included (Data of baseline imbalance is detailed in Table 2). Therefore we carried out summarized baseline analyses for all studies included in this meta-analysis, and found 5 major imbalanced factors: number of liver metastases, size of liver metastases, distribution of liver metastases, scope of hepatectomy and primary tumor location. The number of liver metastases in studies included was mainly reported as “Single vs. Multiple” or “≤3 vs. >3” (Details in Figure 4A). The size of metastases was mainly reported as “≤5 cm vs. >5 cm” or “difference of mean diameter” (Details in Figure 4B). The distribution of metastases was reported as “unilobar vs. bilobar” (Details in Figure 4C). The scope of hepatectomy was reported as “minor hepatectomy vs. major hepatectomy” (Details in Figure 4D). The primary tumor location was reported as “right-sided vs. left-sided vs. rectum” (Details in Figure 4E) or “colon vs. rectum”. The summarized baseline analyses showed that patients were more likely to undergo simultaneous resection when they had lower number of liver metastases (Single metastasis, P = 0.002; ≤3 metastases, P<0.0001), smaller size of liver metastases (diameter ≤5 cm, P = 0.04; smaller mean diameter, P<0.00001), unilobar distribution of liver metastases (P = 0.0002), and need only minor hepatectomy for radical resection (P<0.00001). In terms of primary tumor located, there was no significant imbalance when baseline was compared as “colon vs. rectum” (10 studies reported, OR = 0.82, 95%CI = [0.59, 1.13], P = 0.22). Also, no significant baseline imbalance was observed in low anterior resection vs. abdominoperineal resection (5 studies reported, OR = 0.66, 95%CI = [0.36, 1.21], P = 0.18). But compared as “right-sided vs. left-sided”, significantly more patients had primary tumor located right-sided when they received simultaneous resections (P = 0.0006). Thus, the advantage of simultaneous resection in reducing postoperative complications should be tested and corrected.

**Table 2:** Baseline imbalance in studies included in the meta-analysis.

	Primary tumor			Liver metastases				
Study	Location	T Stage	N Stage	Number	Maximum diameter	Bilobar distribution	Major hepatectomy	Preoperative chemotherapy
**Total number of studies with baseline imbalance**	**3**	**1**	**0**	**12**	**9**	**5**	**11**	**6**
Abbott 2012	N^a^	N	N	Y	NA	NA	Y	N
Alexandrescu 2012	N^b^	NA	NA	N^c^	N^f^	NA	N	Y
Brouquet 2010	N^b^	NA	N	N^e^	Y^g^	Y	Y	NA
Capussotti 2007	N^b^	NA	N	N^c^	N^f^	N	Y	NA
Chua 2004	N^a^	NA	NA	N^c^	N^g^	N	Y	NA
de Haas 2010	N^a^	N	N	Y^c^	N^g^	N	NA	Y
Hu 2013	N^b^	NA	N	N^c^	NA	N	N	NA
Luo 2010	N^b^	Y	N	Y^c^	NA	NA	Y	Y
Martin 2003	Y^a^	NA	NA	Y^c^	Y^f^	NA	Y	NA
Martin 2009	N^a^	NA	N	N^e^	Y^g^	NA	N	Y
Mayo 2013	N^b^	NA	NA	N^c^	Y^g^	N	Y	N
Moug 2010	N^a^	N	N	N	NA	NA	N	N
Reddy 2007	N^b^	N	N	Y^c^	Y^g^	NA	Y	Y
Slupski 2009	N^a^	N	N	Y^e^	Y^g^	NA	N	N
Tanaka 2004	N^b^	N	N	Y^e^	Y^g^	Y	Y	N
Thelen 2007	Y^a^	N	N	Y^d^	N^f^	N	Y	Y
Turrini 2007	N^b^	N	N	Y^d^	NA	Y	NA	NA
Vassiliou 2007	Y^a^	NA	NA	Y^d^	N	N	N	NA
Wang 2008	N^b^	N	N	N^d^	N^f^	Y	NA	NA
Weber 2003	N^a^	N	N	Y^d^	N^f^	N	Y	NA
Xu 2009	N^a^	N	N	N^d^	Y^f^	N	N	NA
Yan 2007	N^a^	NA	N	Y^d^	Y^g^	Y	N	NA

Y: significant imbalance of baseline; N: no significant imbalance of baseline; NA: data not available.

Location was compared as: (right-sided vs. left-sided vs. rectum)^a^ or (colon vs. rectum)^b^. When compared as (right-sided vs. left-sided vs. rectum)^a^, the transverse colon was included in the right-sided, the sigmoid colon was included in the left-sided.

T stage was compared as T1+T2 vs. T3+T4.

N stage was compared as N0 vs. N+.

Number of metastases was compared as: (single vs. multiple)^c^ or (≤3 vs. >3)^d^ or (mean ± SD)^e^ or others with no superscript.

Maximum diameter of metastases was compared as: (≤5 cm vs. >5 cm)^f^ or (mean ± SD)^g^ or others with no superscript.

Major hepatectomy was defined as resection with ≥3 segments.

Preoperative chemotherapy included chemotherapy before both primary resection and hepatectomy.

The study Vassiliou 2007 enrolled only patients with ≤3 liver metastases.

**Figure 5:**
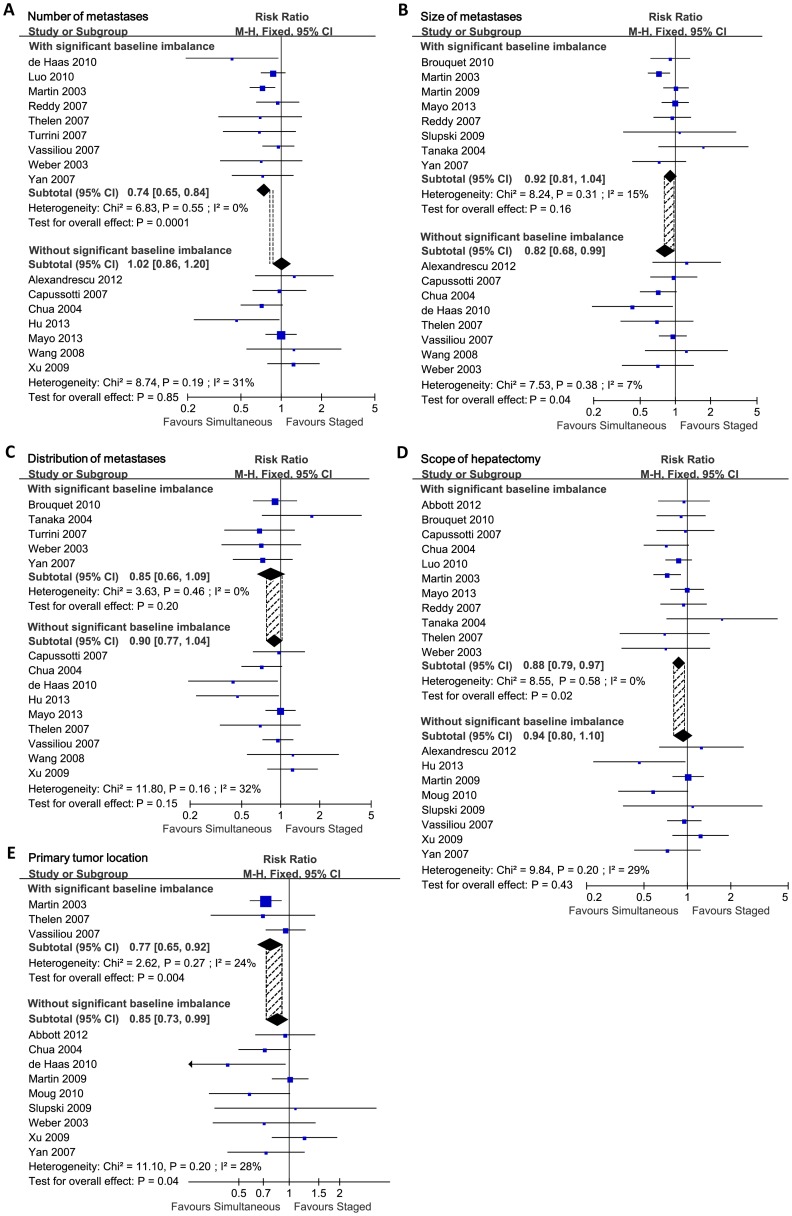
Subgroup analyses in terms of postoperative morbidity. Forest plots displayed the subgroup analyses in terms of postoperative morbidity. Studies with significant baseline imbalances were compared with studies without baseline imbalances. The shaded area between vertical dotted lines represented the overlap regions of the 95% confidence interval of the pooled results between each subgroup. M-H: Mantel-Haenszel method. Fixed: The heterogeneity test showed no significant heterogeneity, and fixed effect model was used. CI: confidence interval. Favour simultaneous/staged: Simultaneous/Staged group had lower postoperative morbidity. **A**) Subgroup analysis on number of liver metastases. **B**) Subgroup analysis on size of liver metastases. **C**) Subgroup analysis on distribution of liver metastases. **D**) Subgroup analysis on scope of hepatectomy. **E**) Subgroup analysis on primary tumor location.

We conducted subgroup analysis of all the 5 imbalanced factors previously mentioned to test the reliability of pooled postoperative morbidity. In terms of the number of liver metastases, the baseline imbalances mainly came from 9 studies, and the other 7 studies had no significant baseline imbalance. Studies with or without significant baseline imbalance were summarized respectively (Details in Figure 5A). By comparing the re-pooled postoperative morbidity of the two subgroups, we found that the re-pooled RRs and 95% confidence interval had significant difference: in the subgroup without baseline imbalance, the pooled morbidity was centered (RR = 1.02, 95%CI = [0.86, 1.21], P = 0.85); but in the subgroup with baseline imbalance, the pooled morbidity was lateralized (RR = 0.74, 95%CI = [0.65, 0.84], P = 0.0001). The 95% confidence interval of two subgroups had no overlap region, which indicated significant differences between the two subgroups. As a confounding factor, number of liver metastases significantly affected the postoperative morbidity when comparing simultaneous and staged strategies. The previously reported advantage of simultaneous resection in reducing complications was not due to the surgical strategy, but due to the lower number of metastases in simultaneous resection group. Actually patients could not benefit more from simultaneous surgical strategy in terms of safety. Without imbalance in number of liver metastases, simultaneous and staged resections were almost the same in postoperative morbidity.

Then, we assessed size of liver metastases, distribution of liver metastases, scope of hepatectomy and primary tumor location (Details in Figure 5). The overlap regions of 95% confidence interval between each subgroup showed that these four factors did not significantly interfere with the postoperative morbidity. The number of liver metastases was the prime factor of selecting surgical strategies. Also, we corrected the baseline imbalances in the subtypes of postoperative complications. After correction, simultaneous resections took significant advantages only in reducing pulmonary complications (RR = 0.23, 95%CI = [0.09, 0.61], P = 0.003). In the other detailed complications (wound infection, anastomotic leakage, bile leakage, subphrenic or perihepatic abscess, cardiac complication, hepatic insufficiency and failure), we failed to observed significant differences between simultaneous and staged groups (Detalis in Figure S6).

Furthermore, we estimated the postoperative morbidity of each subgroup with different number of liver metastases (Details in Information S2). In staged resection group, the morbidity was 13.8% with ≤3 metastases (95%CI = [0–28.6%]), and was 50.9% with >3 metastases (95%CI = [33.8%–67.9%]). In simultaneous resection group, the postoperative morbidity was 17.2% with ≤3 metastases (95%CI = [0–37.3%]), and was 49.4% with >3 metastases (95%CI = [9.4%–89.5%]). For patients received staged resections, the number of metastases was a significant risk factor of postoperative morbidity (P = 0.011). However, for patients received simultaneous resections, the influence of number of metastases was not so significant (P = 0.142). Details were listed in Table 3.

**Table 3:** Postoperative morbidity with different number of liver metastases.

	Simultaneous resection		Staged resection	
Number of liver metastases	Morbidity	95% CI	Morbidity	95% CI
≤3	17.2%	0–37.3%	13.8%	0–28.6%
>3	49.4%	9.4%–89.5%	50.9%	33.8%–67.9%

CI: confidence interval.

Subgroup analyses were also conducted to correct the pooled postoperative mortality and overall survival, and showed that the 5 imbalanced factors previously mentioned did not interfere with these two outcomes. The pooled postoperative mortality and overall survival were credible.

### Preoperative chemotherapy

Preoperative chemotherapy was also one of the possible factors for the short-term and long-term outcomes of SCRLM. The baseline analysis showed that significantly more patients in staged group received preoperative chemotherapy (OR = 0.28, P = 0.004, details in Figure S7). The subgroup analysis showed that the baseline imbalance of preoperative chemotherapy did not interfere with the postoperative morbidity (Details in Figure S8). However, only 6 studies [6], [8], [9], [19], [22], [23] reported both preoperative chemotherapy and long-term survival after operations. Because of lacking of essential data, we were unable to correct the baseline imbalance of preoperative chemotherapy for long-term survival. The results of univariate and multivariate analyses of all studies were listed in Table S2.

### Sensitivity analysis and publication bias test

Sensitivity analyses were performed by consecutively omitting every study from the meta-analysis (leave-one-out procedure). The results were all consistent with each other, indicating the strong robustness of the current study (Details in Table S3).

Publication bias was tested using funnel plots. The funnel plots were symmetrical, similar to inverted funnels, which meant little publication bias existed in this meta-analysis for primary measures (Details in Figure S9).

## Discussion

Without correction of baseline imbalances, pooled results of this meta-analysis showed that for patients with SCRLM, simultaneous resection seemed to have lower postoperative morbidity than staged strategy. In terms of postoperative mortality and long-term survival, simultaneous and staged strategies were similar. In addition, simultaneous strategy could significantly reduce the length of hospital stay, and would not increase the number of patients requiring blood transfusion. These results were consistent with several meta-analyses previously published [10]–[12].

However, we must recognize that many confounding factors also influenced the safety and efficacy of the surgery, just like the number of metastases, the size of metastases, the distribution of metastases, and the scope of hepatectomy. Through baseline analyses we found significant baseline imbalances within studies included: patients in simultaneous groups had much milder conditions than in staged groups. We considered that the advantages of simultaneous strategy in reducing postoperative complications were due to the milder conditions of patients in simultaneous group, but not the surgical procedure itself. And we conducted subgroup analyses to confirm this hypothesis. After the subgroup analyses of imbalanced factors, we found that number of liver metastases was the most significant impact factor for surgical strategy selection. The significantly lower number of metastases in simultaneous resection group, not the surgical strategies, caused the advantages in reducing postoperative morbidity in previously published articles. After baseline correction, simultaneous took no statistical significant advantage in total postoperative morbidity. At the same time, the size and distribution of metastases, the scope of hepatectomy, and the primary tumor location did not significantly interfere with the selection of surgical strategy.

Although there was no difference between the two surgical strategies in total postoperative morbidity, simultaneous resections significantly reduced pulmonary complications, mostly pneumonia and pleuritis. We considered this advantage associated with reducing bedridden time and avoiding a second inhalation anesthesia. In staged resection, a second hepatectomy required another endotracheal intubation and inhalation anesthesia with mechanical ventilation. This also led to additional recovering time in bed, which were risk factors of hospital acquired pneumonia [24], [25].

Then, we attempted to estimate postoperative morbidity of each subgroup with different number of liver metastases. With lower (number ≤3) and higher (number >3) number of liver metastases, the postoperative morbidity in simultaneous and staged resection groups both had no significant difference. But the two surgical approaches were not the same: in staged resection group, higher number of liver metastases was significantly associated with higher postoperative morbidity (P = 0.011); but in simultaneous group, the relevance was not significant (P = 0.142). For simultaneous resections, there might be other confounding factors not detected in our meta-analysis. More data were need to validate and explain these results.

Simultaneous resections were always considered to avoid missing the surgical opportunity. For patients received staged resections, liver metastases may progress during the interval between primary tumor resection and hepatectomy, which would result in missing the opportunity for curative surgery. Simultaneous strategy can avoid this defect. However, this benefit was neglected by most retrospective studies included in this meta-analysis. In these studies, staged group only enrolled patients received successful staged resection. Patients were excluded if their liver metastases progressed and became unresectable during the interval. Thus the OS of staged group was greatly improved. With this in mind, future studies should include patients with progressed liver metastases and missing the secondary hepatectomy to correct this selection bias.

Preoperative neoadjuvant chemotherapy is also one of the factors affecting long-term survival of patients with resectable SCRLM. Because of lacking of data, we were unable to assess the role of preoperative chemotherapy on long-term survival. As a remedy, we summarized the results of univariate and multivariate analyses of all studies included in Table S2. In univariate analyses, only one study considered preoperative chemotherapy as a significant protect factor for liver recurrence after hepatectomy (de Haas et al. [26] P = 0.015). And in multivariate analyses, no study reported preoperative chemotherapy as an independent predictor for long-term survival. However, the influence of this factor displayed mainly in DFS/PFS (progress-free survival), which were reported infrequently in retrospective studies. Currently only one large randomized controlled trial, EORTC 40983 [27], reported perioperative chemotherapy for resectable colorectal liver metastases. The results showed no significant difference between the experimental group and control group in OS (HR = 0.88, P = 0.34). Only eligible patients receiving perioperative chemotherapy had longer PFS after hepatectomy (HR = 0.78, P = 0.035). Considering the primary endpoint of the study was designed as PFS benefit, this result was reasonable. However, it must be noted that study EORTC 40983 compared the perioperative chemotherapy, including both preoperative and postoperative chemotherapy. It was hard to identify the benefits from preoperative chemotherapy.

The limitations of this meta-analysis must be taken into consideration when interpreting its results. Meta-analysis of retrospective studies takes an important part in evidence-based medicine. But compared with RCTs, retrospective studies lack randomization. The comparability between experimental and control group is often poor, and the baseline imbalances significantly affect the accuracy of the pooled results. The method of meta-analysis can only improve the precision of results, not the accuracy. With widespread bias among studies included, meta-analysis is unable to correct errors, even makes the errors more “credible”. Therefore, the population baseline of studies should be tested before pooled analyses. In our meta-analysis, the baseline imbalances were corrected, but not in the best method. The ideal method to correct the confounding factors and establish the selection criteria would be based on the individual patient data analysis (IPD meta-analysis). However, this is not always available, and the diverse reporting forms from the included studies could reduce the reliability of the conclusion. Therefore, the pooled results should be interpret and applied in the most cautious attitude.

At the same time, all studies included in our meta-analysis lacked blinding. Surgeons might pay more attention to patients who received simultaneous resections because of the traditional opinions. These patients might get more strict preoperative preparation and more elaborate postoperative care, which would reduce the postoperative death and complications. Additionally, publication bias was also important. Although the funnel plots suggested no significant presence of publication bias, the impact of bias is always inevitable.

## Conclusions

This meta-analysis was conducted at an appropriate time because simultaneous resection strategy for patients with SCRLM is used more commonly, and enough data has accumulated for pooled analyses. From the pooled analyses and baseline correction, simultaneous resection was as efficient as staged strategy for long-term outcomes, and took an advantage in reducing length of hospital stay. However, it should be emphasized that simultaneous resections took no advantages in reducing postoperative morbidity. Further studies should pay more attention on the number of metastases, which played a significant role in selecting surgical strategies. The size and distribution of liver metastases, the scope of hepatectomy and the primary tumor location did not significantly affect the selection of surgical strategies. Considering the inherent limitations of this meta-analysis, the results should be interpret and applied prudently.

## Supporting Information

Figure S1 Pooled postoperative mortality.
(PDF)

Figure S2 Subtype analysis of pooled postoperative morbidity.
(PDF)

Figure S3 Pooled disease-free survival.
(PDF)

Figure S4 Pooled number of patients need blood transfusion.
(PDF)

Figure S5 Pooled length of hospital stay.
(PDF)

Figure S6 Forest plots of the corrected subtype analysis of postoperative morbidity.
(PDF)

Figure S7 Baseline imbalance of preoperative chemotherapy.
(PDF)

Figure S8 Subgroup analysis of postoperative morbidity on preoperative chemotherapy.
(PDF)

Figure S9 Publication Bias Test.
(PDF)

Table S1 Quality assessment of studies included in the meta-analysis.
(PDF)

Table S2 Summarized prognostic factors for SCRLM within studies included.
(PDF)

Table S3 Sensitivity analysis of postoperative mortality, morbidity and overall survival.
(PDF)

Checklist S1 PRISMA (Preferred Reporting Items for Systematic Reviews and Meta-Analyses) 2009 Checklist.
(DOC)

Information S1 Search strategy for “PubMed”, “Web of Science” and “Embase”.
(PDF)

Information S2 Correction of baseline imbalance in terms of number of liver metastases.
(PDF)

## References

[1] Siegel R, Ma J, Zou Z, Jemal A (2014) Cancer Statistics, 2014. CA Cancer J Clin 10.3322/caac.21208 24399786

[2] Jemal A, Bray F, Center MM, Ferlay J, Ward E, et al (2011) Global cancer statistics. CA Cancer J Clin 61: 69–90. 21296855 10.3322/caac.20107

[3] Poston GJ (2004) Surgical strategies for colorectal liver metastases. Surg Oncol 13: 125–136. 15572095 10.1016/j.suronc.2004.08.001

[4] Altendorf-Hofmann A, Scheele J (2003) A critical review of the major indicators of prognosis after resection of hepatic metastases from colorectal carcinoma. Surg Oncol Clin N Am 12: 165–xi, 165-192, xi. 12735137 10.1016/s1055-3207(02)00091-1

[5] Leporrier J, Maurel J, Chiche L, Bara S, Segol P, et al (2006) A population-based study of the incidence, management and prognosis of hepatic metastases from colorectal cancer. Br J Surg 93: 465–474. 16523446 10.1002/bjs.5278

[6] Alexandrescu S, Hrehoret D, Ionel Z, Croitoru A, Anghel R, et al (2012) Simultaneous resection of the primary colorectal tumor and liver metastases–a safe and effective operation. Chirurgia (Bucur) 107: 298–307. 22844827

[7] Brouquet A, Mortenson MM, Vauthey JN, Rodriguez-Bigas MA, Overman MJ, et al (2010) Surgical strategies for synchronous colorectal liver metastases in 156 consecutive patients: classic, combined or reverse strategy? J Am Coll Surg 210: 934–941. 20510802 10.1016/j.jamcollsurg.2010.02.039

[8] Slupski M, Wlodarczyk Z, Jasinski M, Masztalerz M, Tujakowski J (2009) Outcomes of simultaneous and delayed resections of synchronous colorectal liver metastases. Can J Surg 52: E241–244. 20011158 PMC2792389

[9] Mayo SC, Pulitano C, Marques H, Lamelas J, Wolfgang CL, et al (2013) Surgical management of patients with synchronous colorectal liver metastasis: a multicenter international analysis. J Am Coll Surg 216: 707–716 discussion 716-708. 23433970 10.1016/j.jamcollsurg.2012.12.029 PMC3994665

[10] Chen J, Li Q, Wang C, Zhu H, Shi Y, et al (2011) Simultaneous vs. staged resection for synchronous colorectal liver metastases: a metaanalysis. Int J Colorectal Dis 26: 191–199. 20669024 10.1007/s00384-010-1018-2

[11] Li ZQ, Liu K, Duan JC, Li Z, Su CQ, et al (2013) Meta-analysis of simultaneous versus staged resection for synchronous colorectal liver metastases. Hepatol Res 43: 72–83. 22971038 10.1111/j.1872-034X.2012.01050.x

[12] Yin Z, Liu C, Chen Y, Bai Y, Shang C, et al (2013) Timing of hepatectomy in resectable synchronous colorectal liver metastases (SCRLM): Simultaneous or delayed? Hepatology 57: 2346–2357. 23359206 10.1002/hep.26283

[13] Stroup DF, Berlin JA, Morton SC, Olkin I, Williamson GD, et al (2000) Meta-analysis of observational studies in epidemiology: a proposal for reporting. Meta-analysis Of Observational Studies in Epidemiology (MOOSE) group. JAMA 283: 2008–2012. 10789670 10.1001/jama.283.15.2008

[14] Jadad AR, Moore RA, Carroll D, Jenkinson C, Reynolds DJ, et al (1996) Assessing the quality of reports of randomized clinical trials: is blinding necessary? Control Clin Trials 17: 1–12. 8721797 10.1016/0197-2456(95)00134-4

[15] Wells G, Shea B, O'Connell D, Peterson J, Welch V, et al The Newcastle-Ottawa Scale (NOS) for assessing the quality of nonrandomised studies in meta-analyses. Available at: http://www.ohri.ca/programs/clinical_epidemiology/nosgen.pdf.

[16] Tierney JF, Stewart LA, Ghersi D, Burdett S, Sydes MR (2007) Practical methods for incorporating summary time-to-event data into meta-analysis. Trials 8: 16. 17555582 10.1186/1745-6215-8-16 PMC1920534

[17] Hozo SP, Djulbegovic B, Hozo I (2005) Estimating the mean and variance from the median, range, and the size of a sample. BMC Med Res Methodol 5: 13. 15840177 10.1186/1471-2288-5-13 PMC1097734

[18] Higgins JP, Green S (2011) Cochrane handbook for systematic reviews of interventions version 5.1.0. March 2011. Cochrane Collaboration Available at: http://www.cochrane-handbook.org.

[19] Moug SJ, Smith D, Leen E, Roxburgh C, Horgan PG (2010) Evidence for a synchronous operative approach in the treatment of colorectal cancer with hepatic metastases: a case matched study. Eur J Surg Oncol 36: 365–370. 20034757 10.1016/j.ejso.2009.11.007

[20] Vigano L, Karoui M, Ferrero A, Tayar C, Cherqui D, et al (2011) Locally advanced mid/low rectal cancer with synchronous liver metastases. World J Surg 35: 2788–2795. 21947493 10.1007/s00268-011-1272-7

[21] van der Pool AE, de Wilt JH, Lalmahomed ZS, Eggermont AM, Ijzermans JN, et al (2010) Optimizing the outcome of surgery in patients with rectal cancer and synchronous liver metastases. Br J Surg 97: 383–390. 20101594 10.1002/bjs.6947

[22] Tanaka K, Shimada H, Matsuo K, Nagano Y, Endo I, et al (2004) Outcome after simultaneous colorectal and hepatic resection for colorectal cancer with synchronous metastases. Surgery 136: 650–659. 15349115 10.1016/j.surg.2004.02.012

[23] Thelen A, Jonas S, Benckert C, Spinelli A, Lopez-Hanninen E, et al (2007) Simultaneous versus staged liver resection of synchronous liver metastases from colorectal cancer. Int J Colorectal Dis 22: 1269–1276. 17318552 10.1007/s00384-007-0286-y

[24] Ricard JD, Conti G, Boucherie M, Hormann C, Poelaert J, et al (2012) A European survey of nosocomial infection control and hospital-acquired pneumonia prevention practices. J Infect 65: 285–291. 22771420 10.1016/j.jinf.2012.06.016

[25] Torres A, Ferrer M, Badia JR (2010) Treatment guidelines and outcomes of hospital-acquired and ventilator-associated pneumonia. Clin Infect Dis 51 Suppl 1: S48–53. 20597672 10.1086/653049

[26] de Haas RJ, Adam R, Wicherts DA, Azoulay D, Bismuth H, et al (2010) Comparison of simultaneous or delayed liver surgery for limited synchronous colorectal metastases. Br J Surg 97: 1279–1289. 20578183 10.1002/bjs.7106

[27] Nordlinger B, Sorbye H, Glimelius B, Poston GJ, Schlag PM, et al (2013) Perioperative FOLFOX4 chemotherapy and surgery versus surgery alone for resectable liver metastases from colorectal cancer (EORTC 40983): long-term results of a randomised, controlled, phase 3 trial. Lancet Oncol 14: 1208–1215. 24120480 10.1016/S1470-2045(13)70447-9

[28] Abbott DE, Cantor SB, Hu CY, Aloia TA, You YN, et al (2012) Optimizing clinical and economic outcomes of surgical therapy for patients with colorectal cancer and synchronous liver metastases. J Am Coll Surg 215: 262–270. 22560316 10.1016/j.jamcollsurg.2012.03.021 PMC3688254

[29] Capussotti L, Vigano L, Ferrero A, Lo Tesoriere R, Ribero D, et al (2007) Timing of resection of liver metastases synchronous to colorectal tumor: proposal of prognosis-based decisional model. Ann Surg Oncol 14: 1143–1150. 17200913 10.1245/s10434-006-9284-5

[30] Chua HK, Sondenaa K, Tsiotos GG, Larson DR, Wolff BG, et al (2004) Concurrent vs. staged colectomy and hepatectomy for primary colorectal cancer with synchronous hepatic metastases. Dis Colon Rectum 47: 1310–1316. 15484344 10.1007/s10350-004-0586-z

[31] Hu JJ, Zhou ZX, Liang JW, Wang Z, Zhou HT, et al (2013) Outcome analysis of simultaneous liver resection for synchronous liver metastases from colorectal cancer. Zhonghua Zhong Liu Za Zhi 35: 63–66. 23648304 10.3760/cma.j.issn.0253-3766.2013.01.014

[32] Luo Y, Wang L, Chen C, Chen D, Huang M, et al (2010) Simultaneous liver and colorectal resections are safe for synchronous colorectal liver metastases. J Gastrointest Surg 14: 1974–1980. 20676791 10.1007/s11605-010-1284-x

[33] Martin R, Paty P, Fong Y, Grace A, Cohen A, et al (2003) Simultaneous liver and colorectal resections are safe for synchronous colorectal liver metastasis. J Am Coll Surg 197: 233–241 discussion 241-232. 12892803 10.1016/S1072-7515(03)00390-9

[34] Martin RC 2nd, Augenstein V, Reuter NP, Scoggins CR, McMasters KM (2009) Simultaneous versus staged resection for synchronous colorectal cancer liver metastases. J Am Coll Surg 208: 842–850 discussion 850-842. 19476847 10.1016/j.jamcollsurg.2009.01.031

[35] Reddy SK, Pawlik TM, Zorzi D, Gleisner AL, Ribero D, et al (2007) Simultaneous resections of colorectal cancer and synchronous liver metastases: a multi-institutional analysis. Ann Surg Oncol 14: 3481–3491. 17805933 10.1245/s10434-007-9522-5

[36] Turrini O, Viret F, Guiramand J, Lelong B, Bege T, et al (2007) Strategies for the treatment of synchronous liver metastasis. Eur J Surg Oncol 33: 735–740. 17400418 10.1016/j.ejso.2007.02.025

[37] Vassiliou I, Arkadopoulos N, Theodosopoulos T, Fragulidis G, Marinis A, et al (2007) Surgical approaches of resectable synchronous colorectal liver metastases: timing considerations. World J Gastroenterol 13: 1431–1434. 17457976 10.3748/wjg.v13.i9.1431 PMC4146929

[38] Wang QX, Xu B, Yan JJ, Zhou FG, Yan YQ (2008) Treatment strategy for synchronous liver metastasis from colorectal cancer. Ai Zheng 27: 748–751. 18606070

[39] Weber JC, Bachellier P, Oussoultzoglou E, Jaeck D (2003) Simultaneous resection of colorectal primary tumour and synchronous liver metastases. Br J Surg 90: 956–962. 12905548 10.1002/bjs.4132

[40] Xu J, Wei Y, Zhong Y, Fan J, Zhou J, et al (2009) Hepatectomy for liver metastasis of colorectal cancer. Int J Colorectal Dis 24: 419–425. 19096855 10.1007/s00384-008-0619-5

[41] Yan TD, Chu F, Black D, King DW, Morris DL (2007) Synchronous resection of colorectal primary cancer and liver metastases. World J Surg 31: 1496–1501. 17534545 10.1007/s00268-007-9085-4

