# Supplementary material for: Timing of Hepatectomy for Resectable Synchronous Colorectal Liver Metastases: For Whom Simultaneous Resection Is More Suitable - A Meta-Analysis
Source: PLoS One. 2014 Aug 5;9(8):e104348. doi: 10.1371/journal.pone.0104348 (PMC4122440; doi:10.1371/journal.pone.0104348)
Supplement: Figure S1 — Pooled postoperative mortality. (PDF) [file pone.0104348.s001.pdf]

Figure S1
Pooled postoperative mortality

Study or Subgroup	Simultaneous Events	Simultaneous Total	Staged Events	Staged Total	Weight	Risk Ratio M-H, Fixed, 95% CI
Abbott 2012	2	60	1	84	2.0%	2.80 [0.26, 30.18]
Alexandrescu 2012	7	117	3	25	12.1%	0.50 [0.14, 1.80]
Brouquet 2010	2	43	2	72	3.7%	1.67 [0.24, 11.46]
Capussotti 2007	1	70	0	57	1.3%	2.45 [0.10, 59.03]
Chua 2004	0	64	0	32		Not estimable
de Haas 2010	0	55	1	173	1.8%	1.04 [0.04, 25.07]
Hu 2013	0	40	0	13		Not estimable
Luo 2010	2	129	6	276	9.4%	0.71 [0.15, 3.49]
Martin 2003	3	134	3	106	8.2%	0.79 [0.16, 3.84]
Martin 2009	0	70	1	160	2.2%	0.76 [0.03, 18.33]
Mayo 2013	9	329	21	647	34.6%	0.84 [0.39, 1.82]
Moug 2010	0	32	0	32		Not estimable
Reddy 2007	4	135	5	475	5.4%	2.81 [0.77, 10.34]
Slupski 2009	0	28	1	61	2.3%	0.71 [0.03, 16.97]
Tanaka 2004	0	39	0	37		Not estimable
Thelen 2007	4	40	2	179	1.8%	8.95 [1.70, 47.18]
Turrini 2007	2	57	3	62	7.0%	0.73 [0.13, 4.18]
Vassiliou 2007	0	25	0	78		Not estimable
Wang 2008	0	37	0	46		Not estimable
Weber 2003	0	35	0	62		Not estimable
Xu 2009	4	96	3	79	8.1%	1.10 [0.25, 4.76]
Yan 2007	0	73	0	30		Not estimable
**Total (95% CI)**		**1708**		**2786**	**100.0%**	**1.14 [0.77, 1.69]**
Total events	40		52			

Heterogeneity: Chi² = 11.82, df = 13 (P = 0.54); I² = 0%
Test for overall effect: Z = 0.64 (P = 0.52)

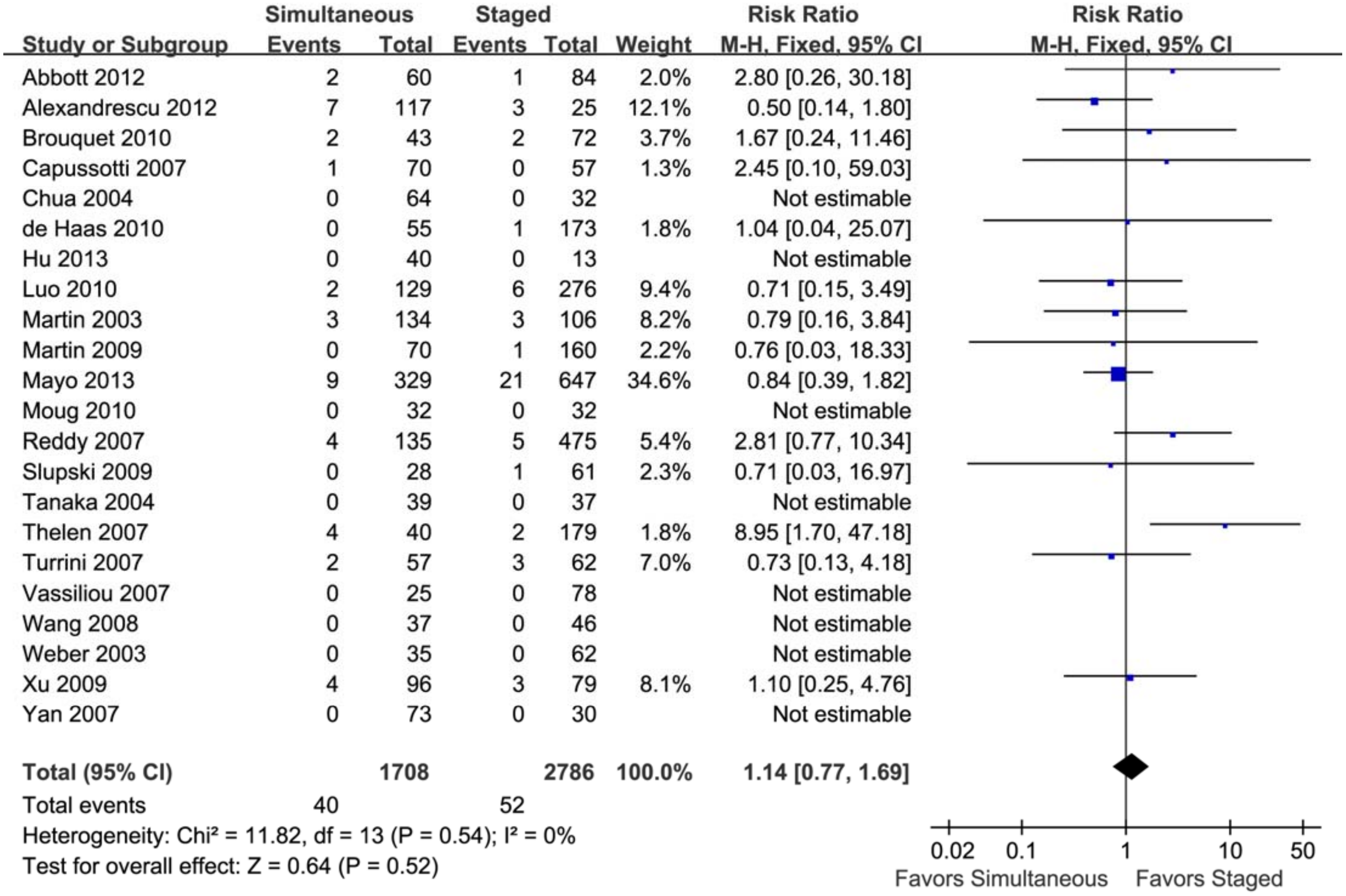

Forest plots of the pooled results on postoperative mortality.
M-H: Mantel-Haenszel method
Favours Simultaneous: Simultaneous group had lower postoperative mortality.
Favours Staged: Staged group had lower postoperative mortality.
Pooled result showed no significant difference between simultaneous and staged groups.
