# Supplementary material for: Timing of Hepatectomy for Resectable Synchronous Colorectal Liver Metastases: For Whom Simultaneous Resection Is More Suitable - A Meta-Analysis
Source: PLoS One. 2014 Aug 5;9(8):e104348. doi: 10.1371/journal.pone.0104348 (PMC4122440; doi:10.1371/journal.pone.0104348)
Supplement: Figure S2 — Subtype analysis of pooled postoperative morbidity. (PDF) [file pone.0104348.s002.pdf]

Figure S2
Subtype analysis of pooled postoperative morbidity

Study or Subgroup	Simultaneous Events	Simultaneous Total	Staged Events	Staged Total	Weight	Risk Ratio M-H, Fixed, 95% CI
**18.1.1 Wound Infection**						
Abbott 2012	2	60	10	84	12.6%	0.28 [0.06, 1.23]
Hu 2013	4	40	3	13	6.9%	0.43 [0.11, 1.69]
Luo 2010	5	129	7	276	6.8%	1.53 [0.49, 4.72]
Martin 2003	17	134	24	106	40.6%	0.56 [0.32, 0.99]
Martin 2009	6	70	8	160	7.4%	1.71 [0.62, 4.76]
Moug 2010	2	32	2	32	3.0%	1.00 [0.15, 6.67]
Thelen 2007	2	40	10	179	5.5%	0.90 [0.20, 3.93]
Vassiliou 2007	2	25	3	78	2.2%	2.08 [0.37, 11.75]
Yan 2007	14	73	7	30	15.0%	0.82 [0.37, 1.83]
**Subtotal (95% CI)**		**603**		**958**	**100.0%**	**0.77 [0.55, 1.08]**
Total events	54		74			
Heterogeneity: Chi² = 8.87, df = 8 (P = 0.35); I² = 10%						
Test for overall effect: Z = 1.52 (P = 0.13)						
**18.1.2 Anastomotic Leakage**						
Abbott 2012	3	60	3	84	19.4%	1.40 [0.29, 6.70]
Alexandrescu 2012	13	117	1	25	12.8%	2.78 [0.38, 20.27]
de Haas 2010	1	55	2	173	7.5%	1.57 [0.15, 17.01]
Luo 2010	4	129	11	276	54.5%	0.78 [0.25, 2.40]
Vassiliou 2007	0	25	1	78	5.8%	1.01 [0.04, 24.11]
**Subtotal (95% CI)**		**386**		**636**	**100.0%**	**1.23 [0.58, 2.59]**
Total events	21		18			
Heterogeneity: Chi² = 1.36, df = 4 (P = 0.85); I² = 0%						
Test for overall effect: Z = 0.54 (P = 0.59)						

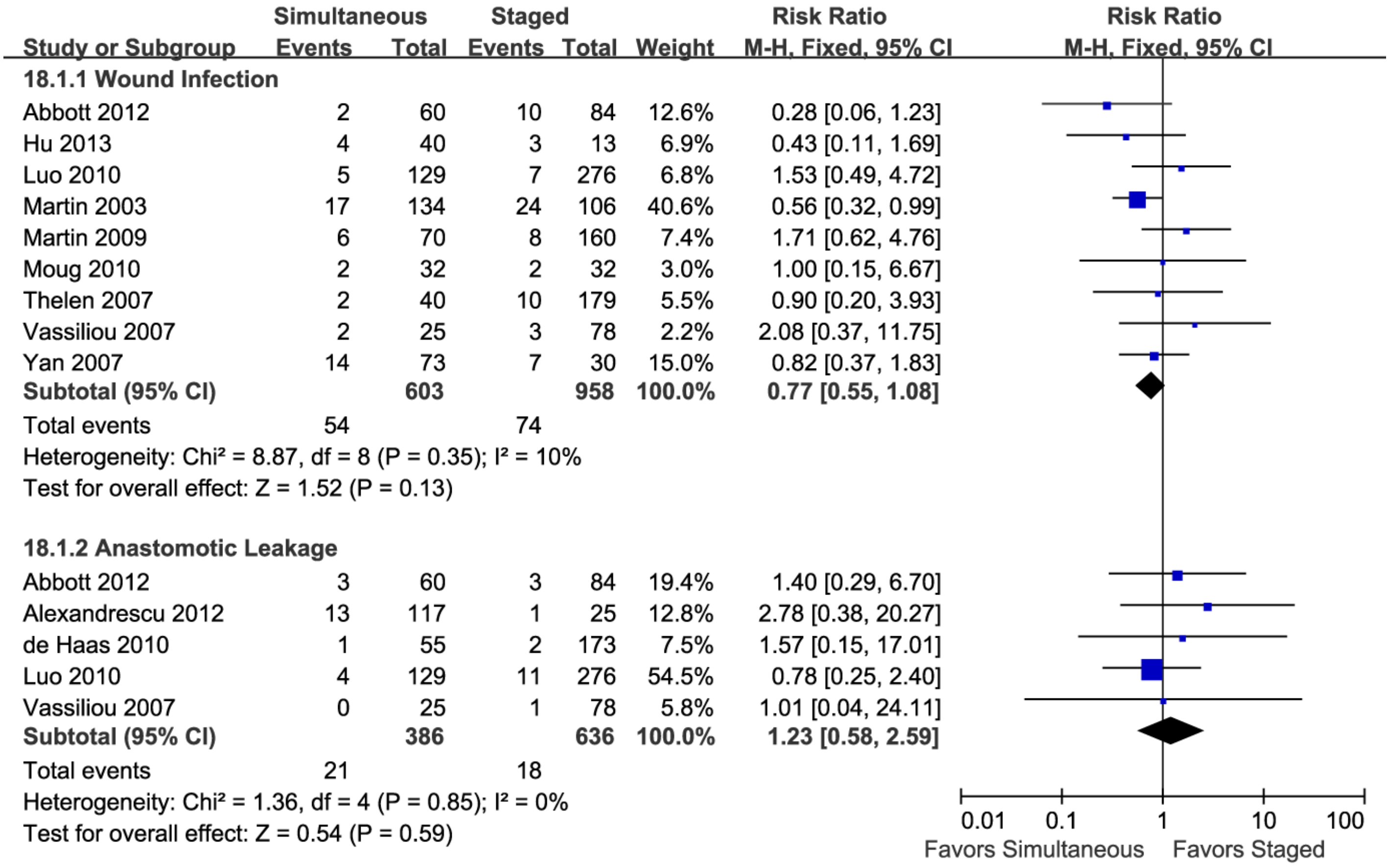

Forest plots on subtype analysis of postoperative morbidity.
M-H: Mantel-Haenszel method
Favours Simultaneous: Simultaneous group had lower postoperative mobidity.
Favours Staged: Staged group had lower postoperative morbidity.
Pooled result showed no significant difference between simultaneous and staged groups in wound infection and anastomotic leakage.

Study or Subgroup	Simultaneous Events	Simultaneous Total	Staged Events	Staged Total	Weight	Risk Ratio M-H, Fixed, 95% CI
**18.1.3 Bile Leakage**						
Abbott 2012	2	60	6	84	13.9%	0.47 [0.10, 2.23]
Alexandrescu 2012	5	117	1	25	4.6%	1.07 [0.13, 8.75]
de Haas 2010	0	55	9	173	12.8%	0.16 [0.01, 2.77]
Hu 2013	3	40	1	13	4.2%	0.97 [0.11, 8.58]
Luo 2010	8	129	21	276	37.1%	0.82 [0.37, 1.79]
Moug 2010	2	32	2	32	5.5%	1.00 [0.15, 6.67]
Tanaka 2004	1	39	3	37	8.5%	0.32 [0.03, 2.91]
Vassiliou 2007	2	25	7	78	9.4%	0.89 [0.20, 4.02]
Yan 2007	1	73	1	30	3.9%	0.41 [0.03, 6.36]
**Subtotal (95% CI)**		**570**		**748**	**100.0%**	**0.66 [0.39, 1.11]**
Total events	24		51			
Heterogeneity: Chi² = 2.60, df = 8 (P = 0.96); I² = 0%						
Test for overall effect: Z = 1.57 (P = 0.12)						
**18.1.4 Subphrenic or Perihepatic Abscess**						
Alexandrescu 2012	13	117	2	25	9.6%	1.39 [0.33, 5.77]
Luo 2010	6	129	7	276	13.1%	1.83 [0.63, 5.35]
Martin 2003	13	134	11	106	36.0%	0.93 [0.44, 2.00]
Martin 2009	6	70	9	160	16.0%	1.52 [0.56, 4.12]
Tanaka 2004	2	39	0	37	1.5%	4.75 [0.24, 95.76]
Vassiliou 2007	3	25	8	78	11.4%	1.17 [0.34, 4.08]
Yan 2007	10	73	3	30	12.4%	1.37 [0.41, 4.63]
**Subtotal (95% CI)**		**587**		**712**	**100.0%**	**1.33 [0.87, 2.02]**
Total events	53		40			
Heterogeneity: Chi² = 1.98, df = 6 (P = 0.92); I² = 0%						
Test for overall effect: Z = 1.32 (P = 0.19)						
**18.1.7 Hepatic Insufficiency and Failure**						
Alexandrescu 2012	4	117	2	25	11.7%	0.43 [0.08, 2.21]
de Haas 2010	0	55	3	173	6.1%	0.44 [0.02, 8.46]
Luo 2010	11	129	17	276	38.5%	1.38 [0.67, 2.87]
Martin 2003	2	134	2	106	7.9%	0.79 [0.11, 5.52]
Martin 2009	1	70	8	160	17.3%	0.29 [0.04, 2.24]
Tanaka 2004	0	39	1	37	5.5%	0.32 [0.01, 7.54]
Thelen 2007	1	40	10	179	13.0%	0.45 [0.06, 3.40]
**Subtotal (95% CI)**		**584**		**956**	**100.0%**	**0.80 [0.46, 1.37]**
Total events	19		43			
Heterogeneity: Chi² = 4.50, df = 6 (P = 0.61); I² = 0%						
Test for overall effect: Z = 0.82 (P = 0.41)						

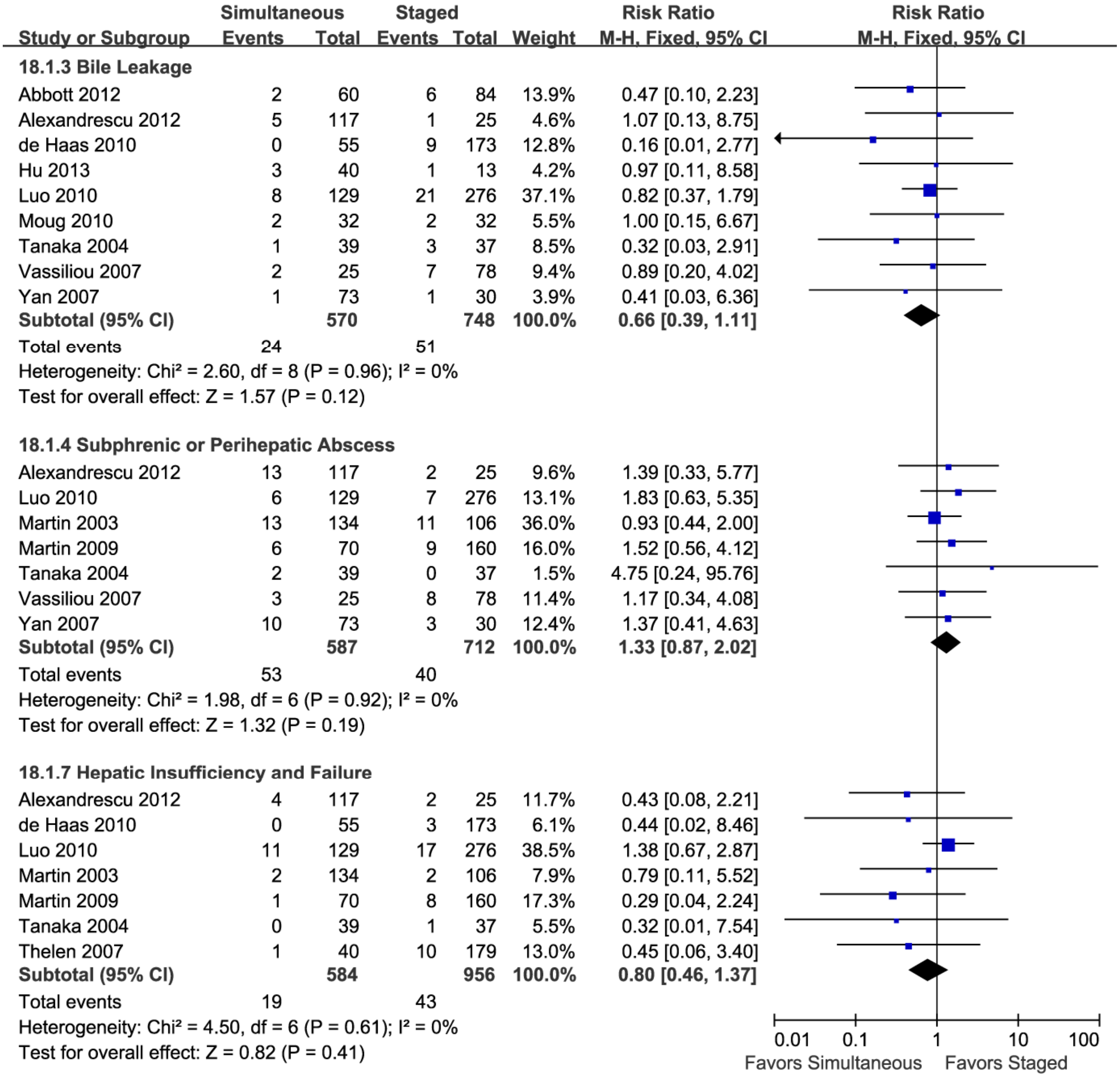

Forest plots on subtype analysis of postoperative morbidity.
M-H: Mantel-Haenszel method
Favours Simultaneous: Simultaneous group had lower postoperative mobidity.
Favours Staged: Staged group had lower postoperative morbidity.
Pooled result showed no significant difference between simultaneous and staged groups in bile leakage, subphrenic/perihepatic abscess and hepatic insufficiency/failure.

Study or Subgroup	Simultaneous Events	Simultaneous Total	Staged Events	Staged Total	Weight	Risk Ratio M-H, Fixed, 95% CI
**18.1.5 Cardiac Complications**						
Abbott 2012	3	60	4	84	12.0%	1.05 [0.24, 4.52]
Martin 2003	5	134	14	106	56.1%	0.28 [0.11, 0.76]
Martin 2009	2	70	11	160	24.0%	0.42 [0.09, 1.83]
Moug 2010	0	32	1	32	5.4%	0.33 [0.01, 7.89]
Yan 2007	1	73	0	30	2.5%	1.26 [0.05, 30.01]
**Subtotal (95% CI)**		**369**		**412**	**100.0%**	**0.43 [0.22, 0.84]**
Total events	11		30			
Heterogeneity: Chi² = 2.59, df = 4 (P = 0.63); I² = 0%						
Test for overall effect: Z = 2.48 (P = 0.01)						
**18.1.6 Pulmonery Complications**						
Abbott 2012	2	60	3	84	3.4%	0.93 [0.16, 5.42]
Alexandrescu 2012	2	117	1	25	2.2%	0.43 [0.04, 4.53]
de Haas 2010	1	55	3	173	2.0%	1.05 [0.11, 9.88]
Hu 2013	2	40	3	13	6.2%	0.22 [0.04, 1.16]
Luo 2010	11	129	18	276	15.6%	1.31 [0.64, 2.69]
Martin 2003	9	134	19	106	28.9%	0.37 [0.18, 0.79]
Martin 2009	2	70	16	160	13.3%	0.29 [0.07, 1.21]
Moug 2010	0	32	5	32	7.5%	0.09 [0.01, 1.58]
Thelen 2007	1	40	4	179	2.0%	1.12 [0.13, 9.74]
Vassiliou 2007	4	25	20	78	13.2%	0.62 [0.24, 1.65]
Yan 2007	5	73	3	30	5.8%	0.68 [0.17, 2.69]
**Subtotal (95% CI)**		**775**		**1156**	**100.0%**	**0.58 [0.40, 0.83]**
Total events	39		95			
Heterogeneity: Chi² = 11.12, df = 10 (P = 0.35); I² = 10%						
Test for overall effect: Z = 3.00 (P = 0.003)						

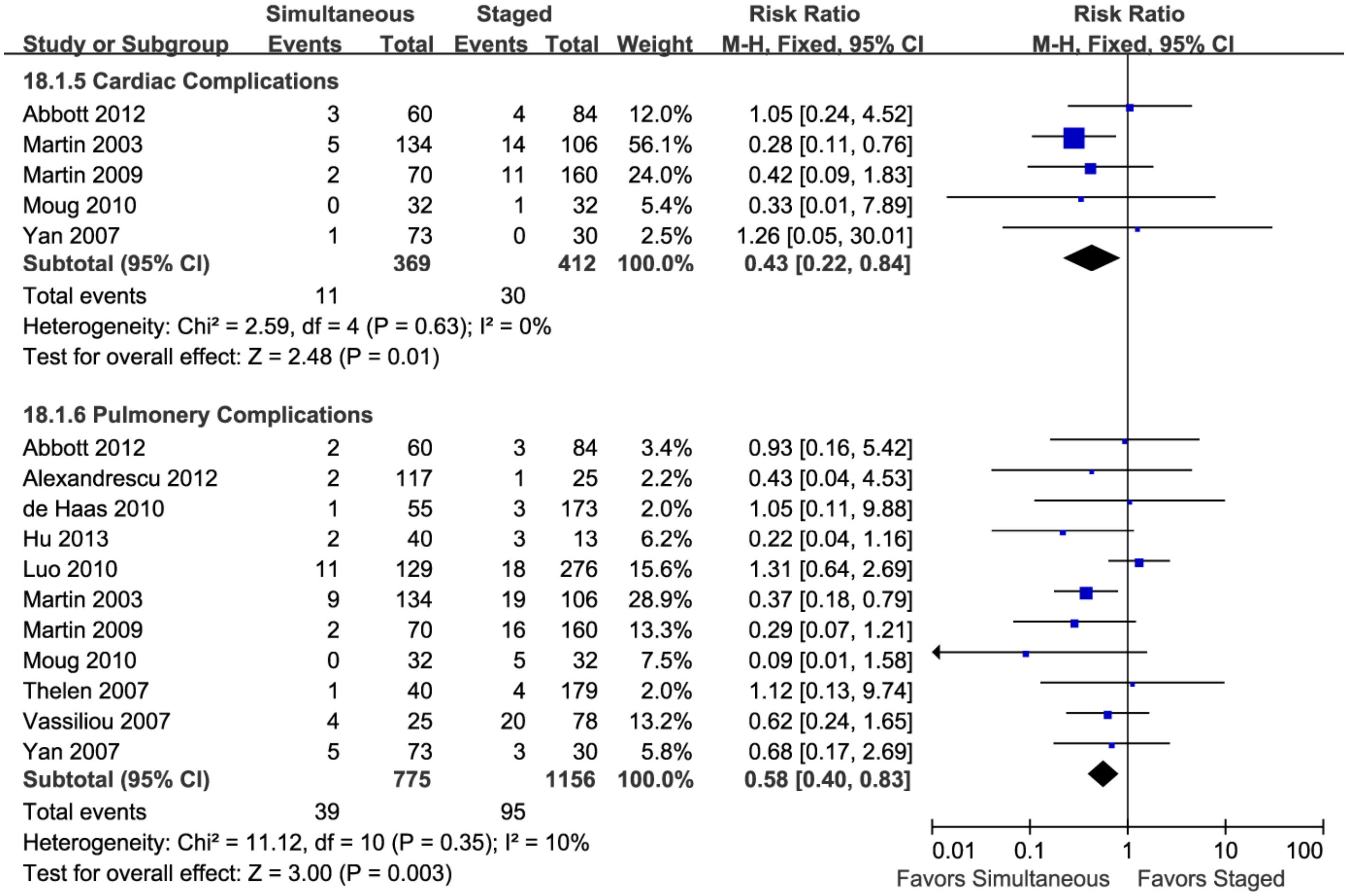

Forest plots on subtype analysis of postoperative morbidity.

M-H: Mantel-Haenszel method

Favours Simultaneous: Simultaneous group had lower postoperative mobidity.

Favours Staged: Staged group had lower postoperative morbidity.

Pooled result showed that simultaneous resection had significant lower morbidity in cardiac and pulmonary complications.
