# Supplementary material for: Timing of Hepatectomy for Resectable Synchronous Colorectal Liver Metastases: For Whom Simultaneous Resection Is More Suitable - A Meta-Analysis
Source: PLoS One. 2014 Aug 5;9(8):e104348. doi: 10.1371/journal.pone.0104348 (PMC4122440; doi:10.1371/journal.pone.0104348)
Supplement: Figure S3 — Pooled disease-free survival. (PDF) [file pone.0104348.s003.pdf]

Figure S3
Pooled disease-free survival

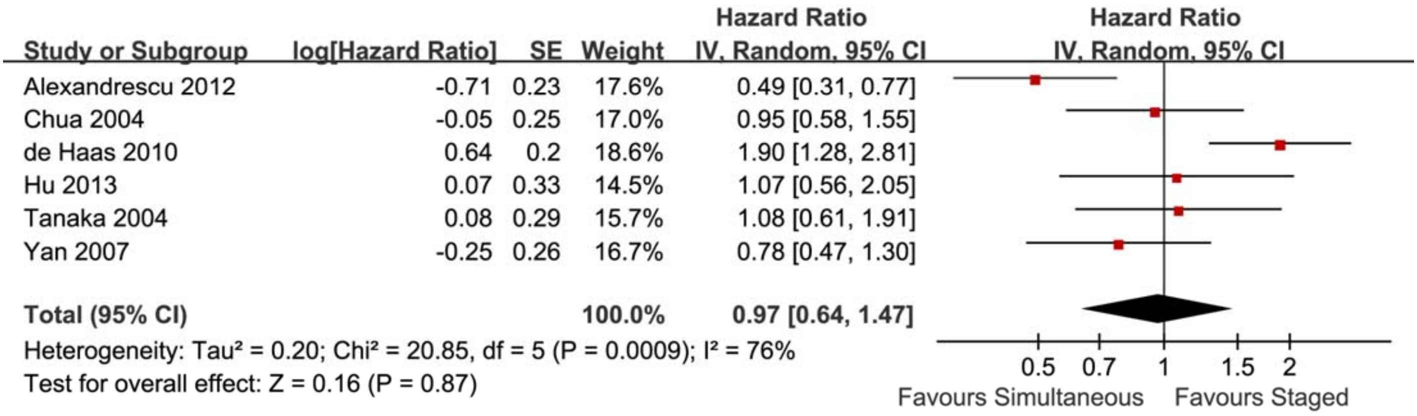

Forest plots of the pooled results on disease-free survival.
Favours Simultaneous: Simultaneous group had longer disease-free survival.
Favours Staged: Staged group had longer disease-free survival.
Pooled result showed no significant difference between simultaneous and staged groups.
