# Supplementary material for: Timing of Hepatectomy for Resectable Synchronous Colorectal Liver Metastases: For Whom Simultaneous Resection Is More Suitable - A Meta-Analysis
Source: PLoS One. 2014 Aug 5;9(8):e104348. doi: 10.1371/journal.pone.0104348 (PMC4122440; doi:10.1371/journal.pone.0104348)
Supplement: Figure S4 — Pooled number of patients need blood transfusion. (PDF) [file pone.0104348.s004.pdf]

Figure S4
Pooled number of patients need blood transfusion

Study or Subgroup	Simultaneous Events	Simultaneous Total	Staged Events	Staged Total	Weight	Risk Ratio M-H, Random, 95% CI
Brouquet 2010	7	43	9	72	4.7%	1.30 [0.52, 3.24]
Capussotti 2007	13	31	8	48	6.4%	2.52 [1.18, 5.36]
Martin 2003	42	134	41	106	17.2%	0.81 [0.57, 1.15]
Martin 2009	35	70	72	160	19.8%	1.11 [0.83, 1.49]
Moug 2010	0	32	1	32	0.5%	0.33 [0.01, 7.89]
Reddy 2007	28	135	72	475	15.3%	1.37 [0.92, 2.03]
Thelen 2007	14	40	38	179	11.4%	1.65 [0.99, 2.74]
Weber 2003	17	35	35	62	14.8%	0.86 [0.57, 1.29]
Xu 2009	22	96	17	79	10.0%	1.06 [0.61, 1.86]
**Total (95% CI)**		**616**		**1213**	**100.0%**	**1.15 [0.93, 1.43]**
Total events	178		293			

Heterogeneity: Tau² = 0.04; Chi² = 13.34, df = 8 (P = 0.10); I² = 40%
Test for overall effect: Z = 1.28 (P = 0.20)

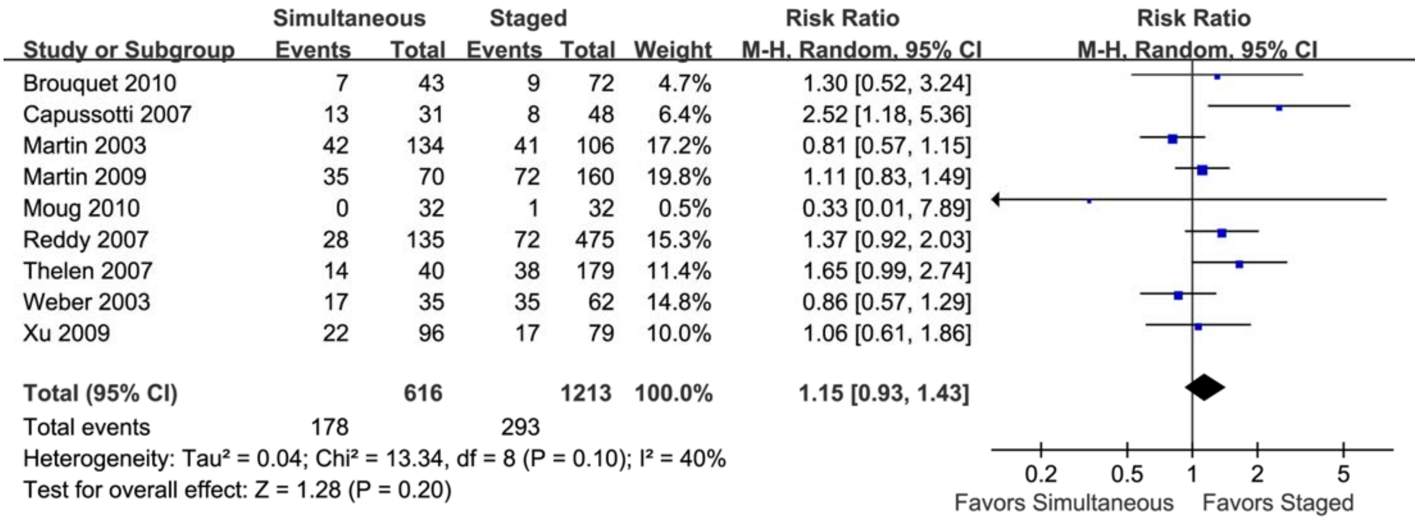

Forest plots of the pooled results on number of patients need blood transfusion.
M-H: Mantel-Haenszel method
Favours Simultaneous: Simultaneous group had fewer patients need blood transfusion.
Favours Staged: Staged group had fewer patients need blood transfusion.
Pooled result showed no significant difference between simultaneous and staged groups.
