# Supplementary material for: Timing of Hepatectomy for Resectable Synchronous Colorectal Liver Metastases: For Whom Simultaneous Resection Is More Suitable - A Meta-Analysis
Source: PLoS One. 2014 Aug 5;9(8):e104348. doi: 10.1371/journal.pone.0104348 (PMC4122440; doi:10.1371/journal.pone.0104348)
Supplement: Figure S5 — Pooled length of hospital stay. (PDF) [file pone.0104348.s005.pdf]

Figure S5
Pooled length of hospital stay

	Simultaneous			Staged				Mean Difference
Study or Subgroup	Mean	SD	Total	Mean	SD	Total	Weight	IV, Random, 95% CI
Abbott 2012	8	3	60	14	4.4	84	10.6%	-6.00 [-7.21, -4.79]
Chua 2004	14.4	6.7	64	22.4	17.6	32	1.7%	-8.00 [-14.32, -1.68]
Hu 2013	16.2	8.1	40	25.8	8.5	13	2.4%	-9.60 [-14.86, -4.34]
Luo 2010	8	1.3	129	14	1.8	276	12.9%	-6.00 [-6.31, -5.69]
Martin 2003	10	8.25	134	18	8.5	106	7.5%	-8.00 [-10.14, -5.86]
Martin 2009	10	14.5	70	18	8	160	4.2%	-8.00 [-11.62, -4.38]
Moug 2010	13.3	3.2	32	24.6	11	32	3.7%	-11.30 [-15.27, -7.33]
Reddy 2007	9	3	135	14	3.7	475	12.4%	-5.00 [-5.61, -4.39]
Slupski 2009	12	4	28	15	3.5	61	8.8%	-3.00 [-4.72, -1.28]
Tanaka 2004	25.6	10.4	39	23.1	10.3	37	2.9%	2.50 [-2.15, 7.15]
Thelen 2007	20.05	8	40	19.85	30.78	179	2.5%	0.20 [-4.95, 5.35]
Vassiliou 2007	12	6	25	20	8	78	5.4%	-8.00 [-10.95, -5.05]
Weber 2003	17	9	35	16	7	62	4.4%	1.00 [-2.45, 4.45]
Xu 2009	10	2	96	15	3	79	11.9%	-5.00 [-5.77, -4.23]
Yan 2007	9.5	2.3	73	16.5	4.5	30	8.9%	-7.00 [-8.69, -5.31]
**Total (95% CI)**			**1000**			**1704**	**100.0%**	**-5.53 [-6.42, -4.64]**

Heterogeneity: $Tau^2 = 1.59$; $Chi^2 = 73.88$, df = 14 ($P < 0.00001$); $I^2 = 81\%$
Test for overall effect: $Z = 12.13$ ($P < 0.00001$)

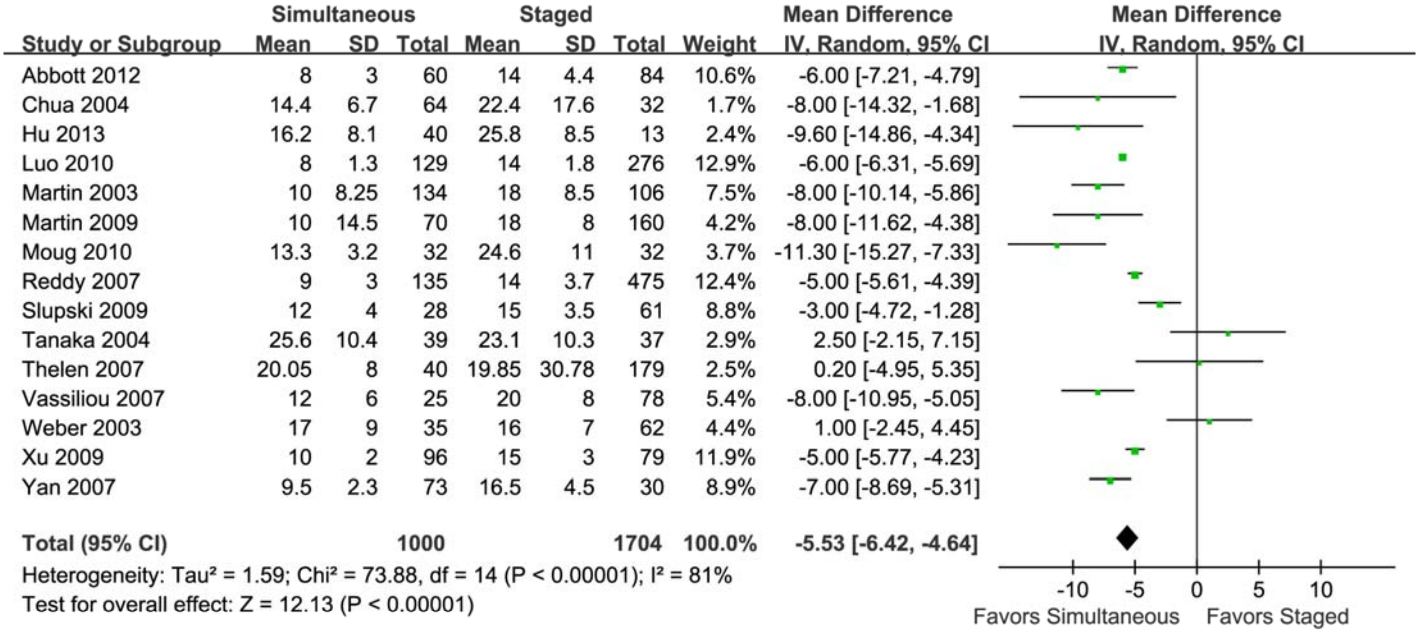

Forest plots of the pooled results on length of hospital stay.
Favours Simultaneous: Simultaneous group had shorter hospital stay.
Favours Staged: Staged group had shorter hospital stay.
Pooled result showed that simultaneous resections significantly reduced the length of hospital stay.
