# Supplementary material for: Timing of Hepatectomy for Resectable Synchronous Colorectal Liver Metastases: For Whom Simultaneous Resection Is More Suitable - A Meta-Analysis
Source: PLoS One. 2014 Aug 5;9(8):e104348. doi: 10.1371/journal.pone.0104348 (PMC4122440; doi:10.1371/journal.pone.0104348)
Supplement: Figure S6 — Forest plots of the corrected subtype analysis of postoperative morbidity. (PDF) [file pone.0104348.s006.pdf]

Figure S6
Corrected subtype analysis of postoperative morbidity

Study or Subgroup	Simultaneous Events	Simultaneous Total	Staged Events	Staged Total	Weight	Risk Ratio M-H, Fixed, 95% CI
**18.2.1 Wound Infection**						
Hu 2013	4	40	3	13	39.7%	0.43 [0.11, 1.69]
Martin 2009	6	70	8	160	42.7%	1.71 [0.62, 4.76]
Moug 2010	2	32	2	32	17.5%	1.00 [0.15, 6.67]
**Subtotal (95% CI)**		**142**		**205**	**100.0%**	**1.08 [0.52, 2.22]**
Total events	12		13			
Heterogeneity: Chi² = 2.53, df = 2 (P = 0.28); I² = 21%						
Test for overall effect: Z = 0.21 (P = 0.83)						
**18.2.2 Anastomotic Leakage**						
Alexandrescu 2012	13	117	1	25	100.0%	2.78 [0.38, 20.27]
**Subtotal (95% CI)**		**117**		**25**	**100.0%**	**2.78 [0.38, 20.27]**
Total events	13		1			
Heterogeneity: Not applicable						
Test for overall effect: Z = 1.01 (P = 0.31)						
**18.2.3 Bile Leakage**						
Alexandrescu 2012	5	117	1	25	32.0%	1.07 [0.13, 8.75]
Hu 2013	3	40	1	13	29.3%	0.97 [0.11, 8.58]
Moug 2010	2	32	2	32	38.8%	1.00 [0.15, 6.67]
**Subtotal (95% CI)**		**189**		**70**	**100.0%**	**1.01 [0.31, 3.31]**
Total events	10		4			
Heterogeneity: Chi² = 0.00, df = 2 (P = 1.00); I² = 0%						
Test for overall effect: Z = 0.02 (P = 0.98)						
**18.2.4 Subphrenic or Perihepatic Abscess**						
Alexandrescu 2012	13	117	2	25	37.6%	1.39 [0.33, 5.77]
Martin 2009	6	70	9	160	62.4%	1.52 [0.56, 4.12]
**Subtotal (95% CI)**		**187**		**185**	**100.0%**	**1.47 [0.65, 3.34]**
Total events	19		11			
Heterogeneity: Chi² = 0.01, df = 1 (P = 0.92); I² = 0%						
Test for overall effect: Z = 0.93 (P = 0.35)						

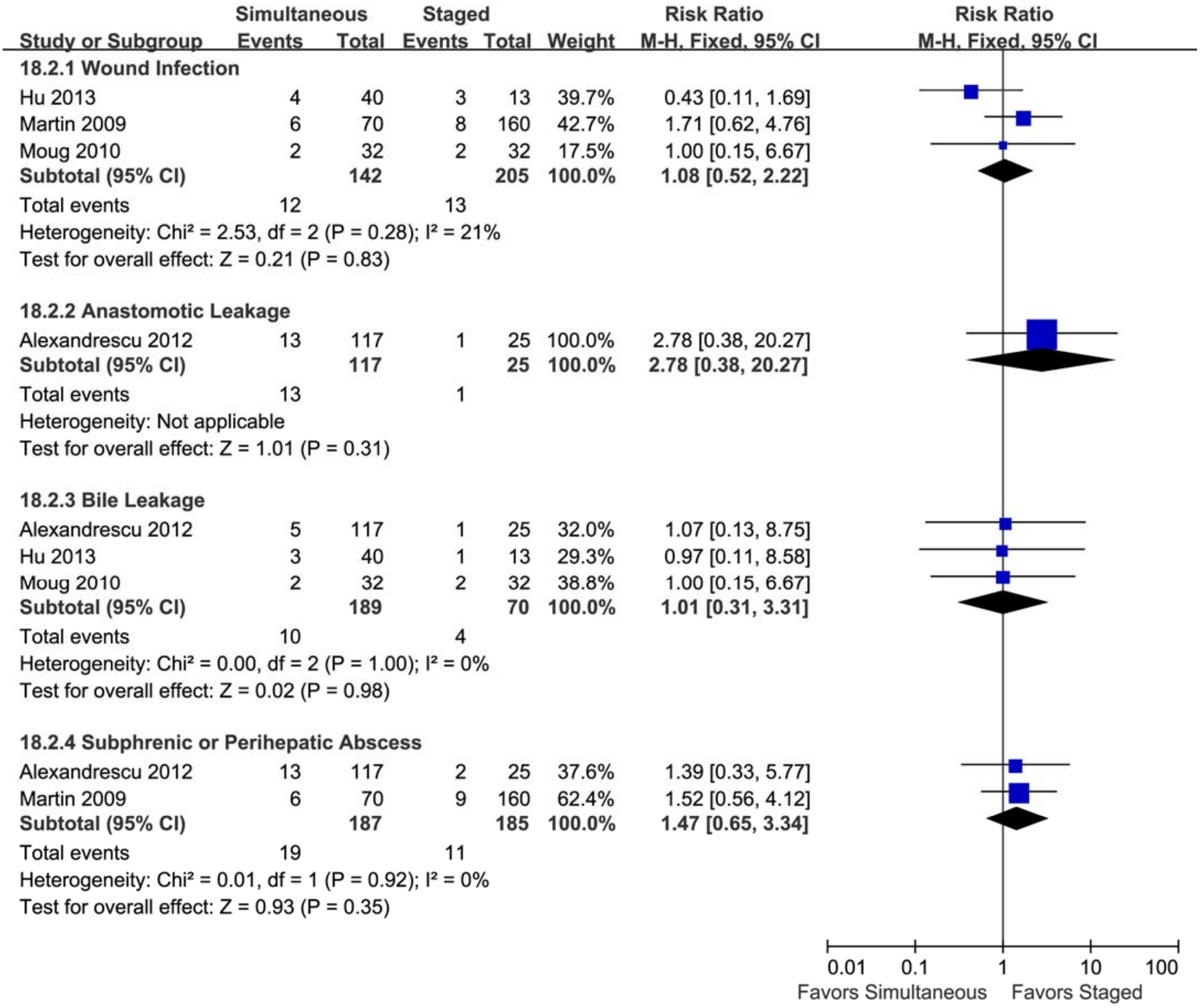

Study or Subgroup	Simultaneous Events	Simultaneous Total	Staged Events	Staged Total	Weight	Risk Ratio M-H, Fixed, 95% CI
**18.2.5 Cardiac Complications**						
Martin 2009	2	70	11	160	81.7%	0.42 [0.09, 1.83]
Moug 2010	0	32	1	32	18.3%	0.33 [0.01, 7.89]
**Subtotal (95% CI)**		**102**		**192**	**100.0%**	**0.40 [0.10, 1.53]**
Total events	2		12			
Heterogeneity: Chi² = 0.02, df = 1 (P = 0.90); I² = 0%						
Test for overall effect: Z = 1.34 (P = 0.18)						
**18.2.6 Pulmonery Complications**						
Alexandrescu 2012	2	117	1	25	7.7%	0.43 [0.04, 4.53]
Hu 2013	2	40	3	13	21.1%	0.22 [0.04, 1.16]
Martin 2009	2	70	16	160	45.5%	0.29 [0.07, 1.21]
Moug 2010	0	32	5	32	25.7%	0.09 [0.01, 1.58]
**Subtotal (95% CI)**		**259**		**230**	**100.0%**	**0.23 [0.09, 0.61]**
Total events	6		25			
Heterogeneity: Chi² = 0.76, df = 3 (P = 0.86); I² = 0%						
Test for overall effect: Z = 2.99 (P = 0.003)						
**18.2.7 Hepatic Insufficiency and Failure**						
Alexandrescu 2012	4	117	2	25	40.4%	0.43 [0.08, 2.21]
Martin 2009	1	70	8	160	59.6%	0.29 [0.04, 2.24]
**Subtotal (95% CI)**		**187**		**185**	**100.0%**	**0.34 [0.09, 1.31]**
Total events	5		10			
Heterogeneity: Chi² = 0.10, df = 1 (P = 0.75); I² = 0%						
Test for overall effect: Z = 1.56 (P = 0.12)						

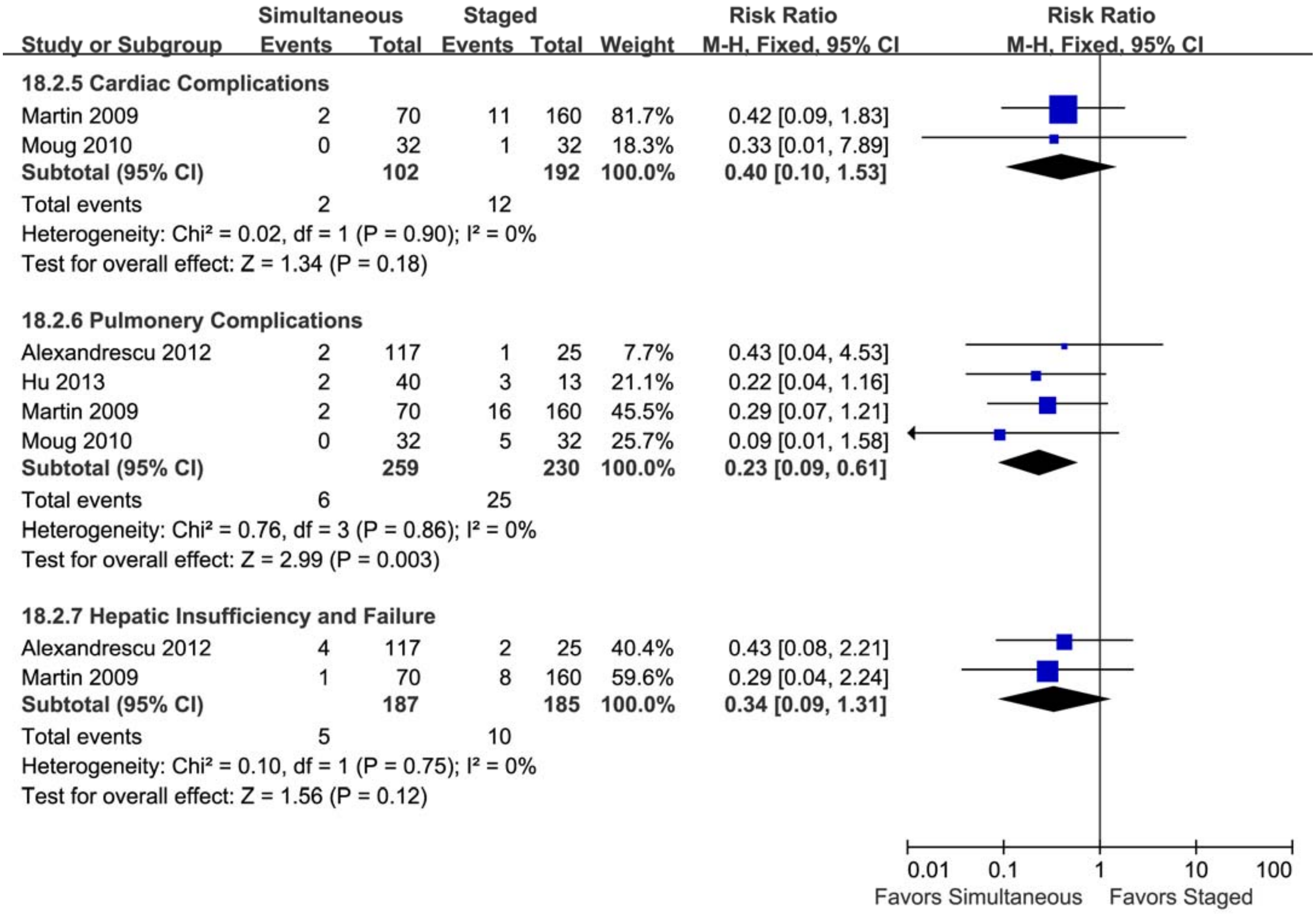

Forest plots of the corrected subtype analysis of postoperative morbidity.

M-H: Mantel-Haenszel method

Favours Simultaneous: Simultaneous group had lower postoperative mobidity.

Favours Staged: Staged group had lower postoperative morbidity.

All results were corrected according to baseline analyses.

Pooled result showed significant difference between simultaneous and staged groups only in pulmonary complications. No other significant difference was observed.
