# Supplementary material for: Timing of Hepatectomy for Resectable Synchronous Colorectal Liver Metastases: For Whom Simultaneous Resection Is More Suitable - A Meta-Analysis
Source: PLoS One. 2014 Aug 5;9(8):e104348. doi: 10.1371/journal.pone.0104348 (PMC4122440; doi:10.1371/journal.pone.0104348)
Supplement: Figure S7 — Baseline imbalance of preoperative chemotherapy. (PDF) [file pone.0104348.s007.pdf]

Figure S7
Baseline imbalance of preoperative chemotherapy

Study or Subgroup	Simultaneous Events	Simultaneous Total	Staged Events	Staged Total	Weight	Odds Ratio M-H, Random, 95% CI
Abbott 2012	46	60	52	84	11.9%	2.02 [0.96, 4.25]
Alexandrescu 2012	12	117	25	25	5.4%	0.00 [0.00, 0.04]
de Haas 2010	13	55	165	173	11.3%	0.02 [0.01, 0.04]
Luo 2010	51	129	169	276	12.7%	0.41 [0.27, 0.64]
Martin 2009	36	70	112	160	12.3%	0.45 [0.25, 0.81]
Mayo 2013	114	329	189	647	12.9%	1.28 [0.97, 1.71]
Moug 2010	13	32	17	32	11.2%	0.60 [0.22, 1.62]
Reddy 2007	82	135	59	70	12.0%	0.29 [0.14, 0.60]
Slupski 2009	0	28	0	61		Not estimable
Tanaka 2004	0	39	0	37		Not estimable
Thelen 2007	3	40	49	179	10.4%	0.22 [0.06, 0.73]
**Total (95% CI)**		**1034**		**1744**	**100.0%**	**0.28 [0.12, 0.68]**
Total events	370		837			

Heterogeneity: Tau² = 1.50; Chi² = 124.02, df = 8 (P < 0.00001); I² = 94%
Test for overall effect: Z = 2.85 (P = 0.004)

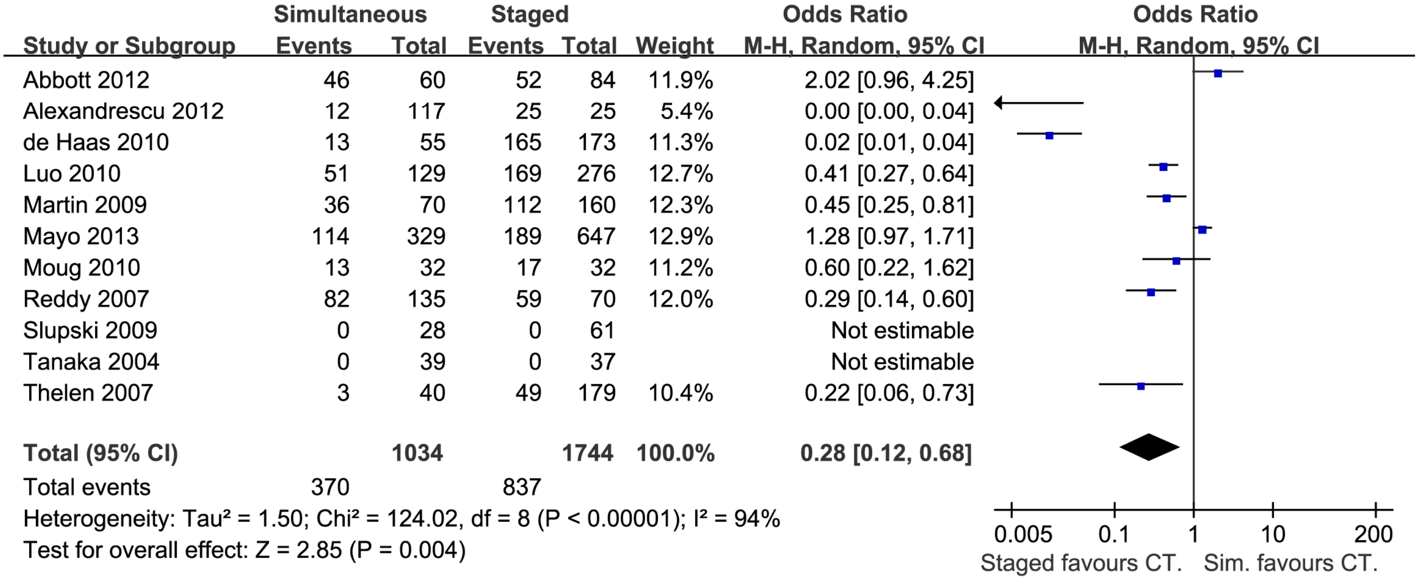

Forest plots of the baseline imbalance of preoperative chemotherapy.
M-H: Mantel-Haenszel method
Staged favours CT.: More patients in staged group received preoperative chemotherapy.
Sim. favours CT.: More patients in simultaneous group received preoperative chemotherapy.
Pooled result showed that significantly more patients in staged group received preoperative chemotherapy. There was significant baseline imbalance of preoperative chemotherapy.
