# Supplementary material for: Timing of Hepatectomy for Resectable Synchronous Colorectal Liver Metastases: For Whom Simultaneous Resection Is More Suitable - A Meta-Analysis
Source: PLoS One. 2014 Aug 5;9(8):e104348. doi: 10.1371/journal.pone.0104348 (PMC4122440; doi:10.1371/journal.pone.0104348)
Supplement: Figure S8 — Subgroup analysis of postoperative morbidity on preoperative chemotherapy. (PDF) [file pone.0104348.s008.pdf]

Figure S8
Subgroup analysis of postoperative morbidity on preoperative chemotherapy

Study or Subgroup	Simultaneous Events	Simultaneous Total	Staged Events	Staged Total	Weight	Odds Ratio M-H, Fixed, 95% CI
**11.1.1 With significant baseline bias**						
Alexandrescu 2012	41	117	7	25	5.5%	1.39 [0.54, 3.59]
de Haas 2010	6	55	44	173	13.8%	0.36 [0.14, 0.90]
Luo 2010	61	129	150	276	36.8%	0.75 [0.50, 1.15]
Martin 2009	39	70	88	160	17.4%	1.03 [0.58, 1.81]
Reddy 2007	49	135	27	70	16.6%	0.91 [0.50, 1.65]
Thelen 2007	7	40	45	179	9.9%	0.63 [0.26, 1.53]
**Subtotal (95% CI)**		**546**		**883**	**100.0%**	**0.79 [0.62, 1.02]**
Total events	203		361			
Heterogeneity: Chi² = 5.53, df = 5 (P = 0.35); I² = 10%						
Test for overall effect: Z = 1.79 (P = 0.07)						
**11.1.2 Without significant baseline bias**						
Abbott 2012	23	60	34	84	16.5%	0.91 [0.46, 1.80]
Mayo 2013	63	303	128	614	63.4%	1.00 [0.71, 1.40]
Moug 2010	11	32	19	32	11.8%	0.36 [0.13, 0.99]
Slupski 2009	4	28	8	61	4.1%	1.10 [0.30, 4.03]
Tanaka 2004	11	39	6	37	4.2%	2.03 [0.66, 6.21]
**Subtotal (95% CI)**		**462**		**828**	**100.0%**	**0.96 [0.73, 1.26]**
Total events	112		195			
Heterogeneity: Chi² = 5.45, df = 4 (P = 0.24); I² = 27%						
Test for overall effect: Z = 0.33 (P = 0.74)						

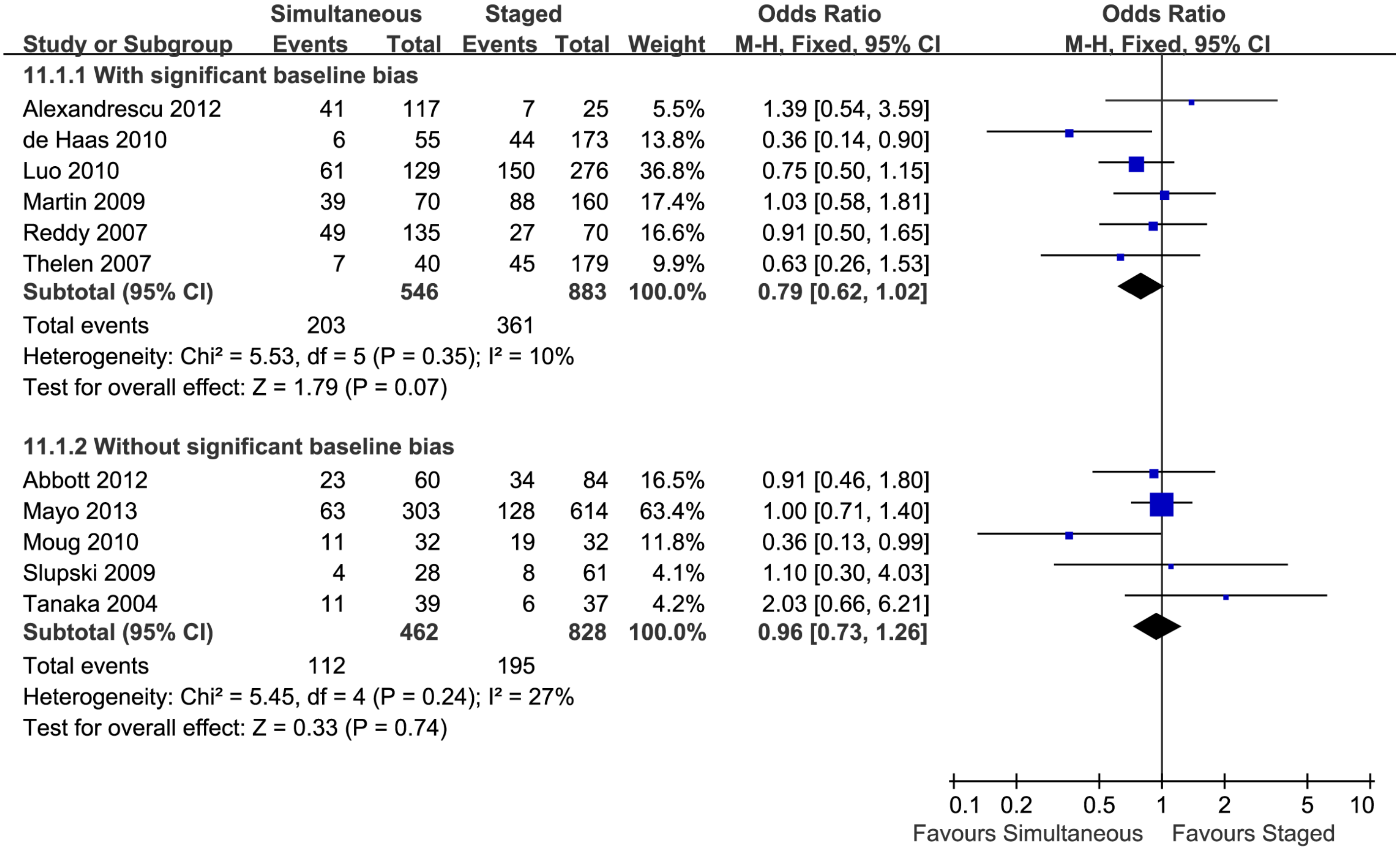

Forest plots of subgroup analysis of postoperative morbidity on preoperative chemotherapy.

Favours Simultaneous: Simultaneous group had lower postoperative morbidity.

Favours Staged: Staged group had lower postoperative morbidity.

The overlap regions of 95% confidence interval between the two subgroups showed that preoperative chemotherapy did not significantly interfere with the postoperative morbidity.
