# Supplementary material for: Timing of Hepatectomy for Resectable Synchronous Colorectal Liver Metastases: For Whom Simultaneous Resection Is More Suitable - A Meta-Analysis
Source: PLoS One. 2014 Aug 5;9(8):e104348. doi: 10.1371/journal.pone.0104348 (PMC4122440; doi:10.1371/journal.pone.0104348)
Supplement: Figure S9 — Publication Bias Test. (PDF) [file pone.0104348.s009.pdf]

Figure S9
Publication Bias Test

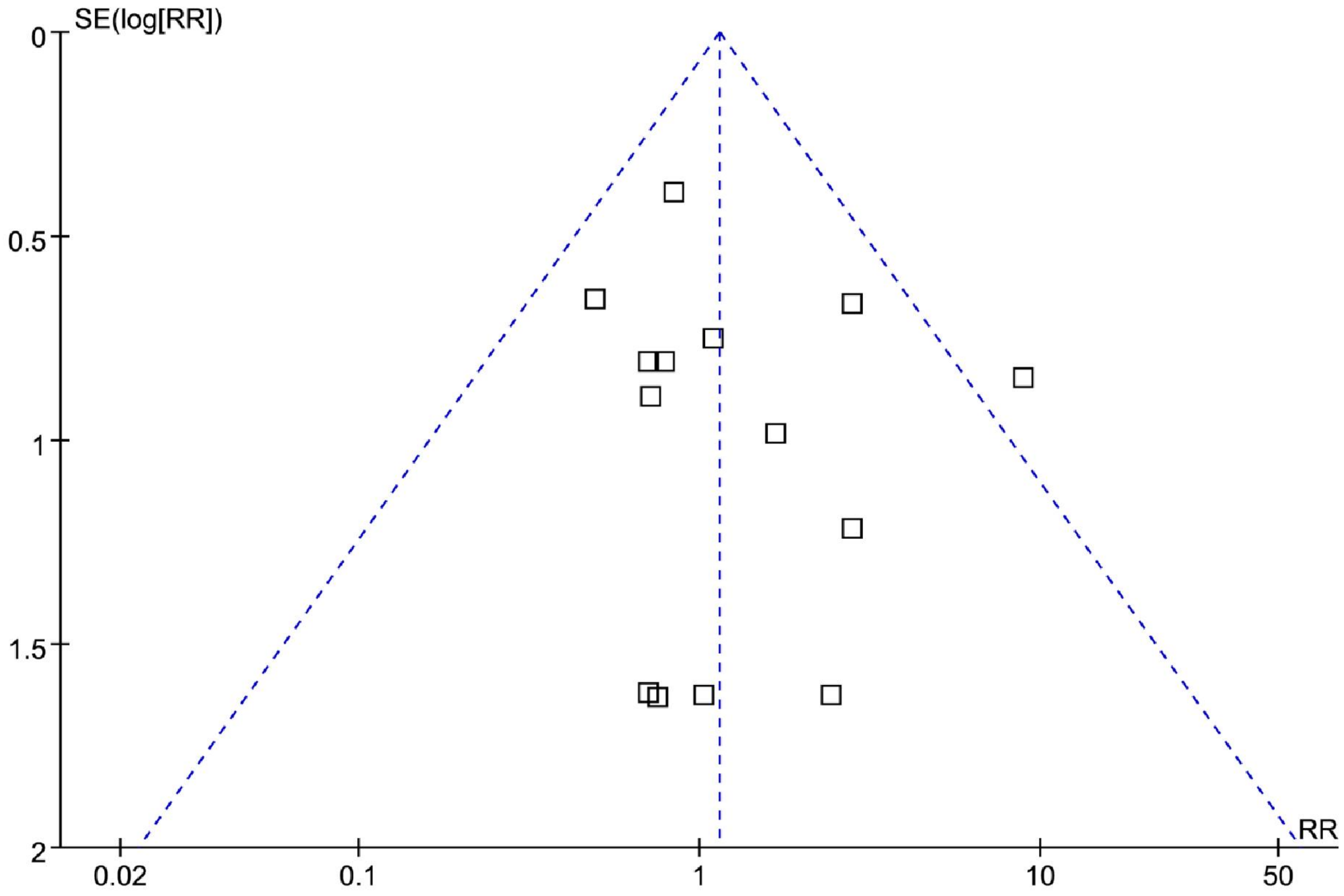

Funnel plot for all studies comparing postoperative mortality.
Each study is marked by a dot around the overall effect estimate (vertical line). The diagonal lines represent the 95% confidence interval around the overall effect estimate. The symmetrical distribution of all dots showed that there was no significant publication bias. RR, risk ratio; SE, standard error.

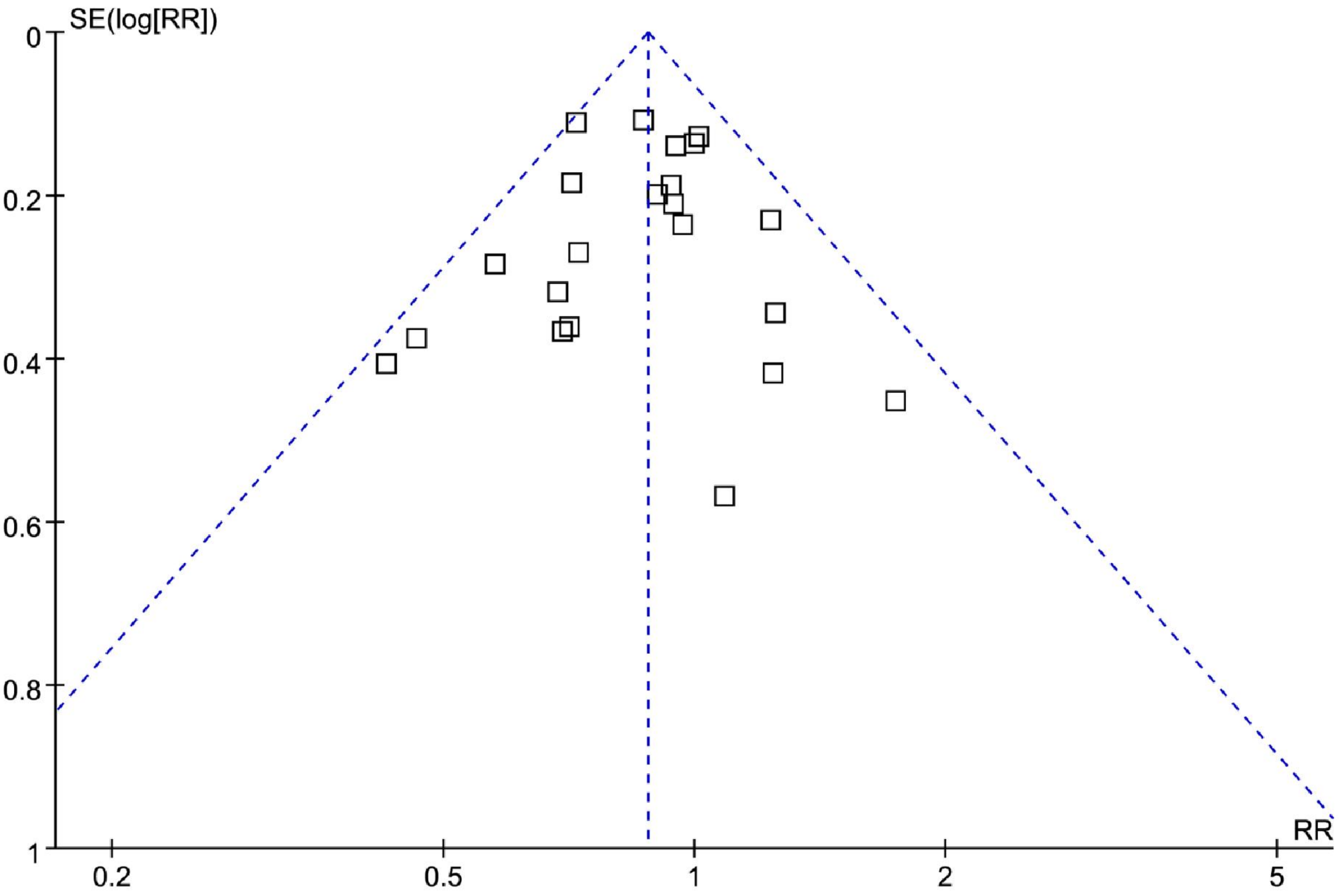

Funnel plot for all studies comparing postoperative morbidity.
Each study is marked by a dot around the overall effect estimate (vertical line). The diagonal lines represent the 95% confidence interval around the overall effect estimate. The symmetrical distribution of all dots showed that there was no significant publication bias. RR, risk ratio; SE, standard error.

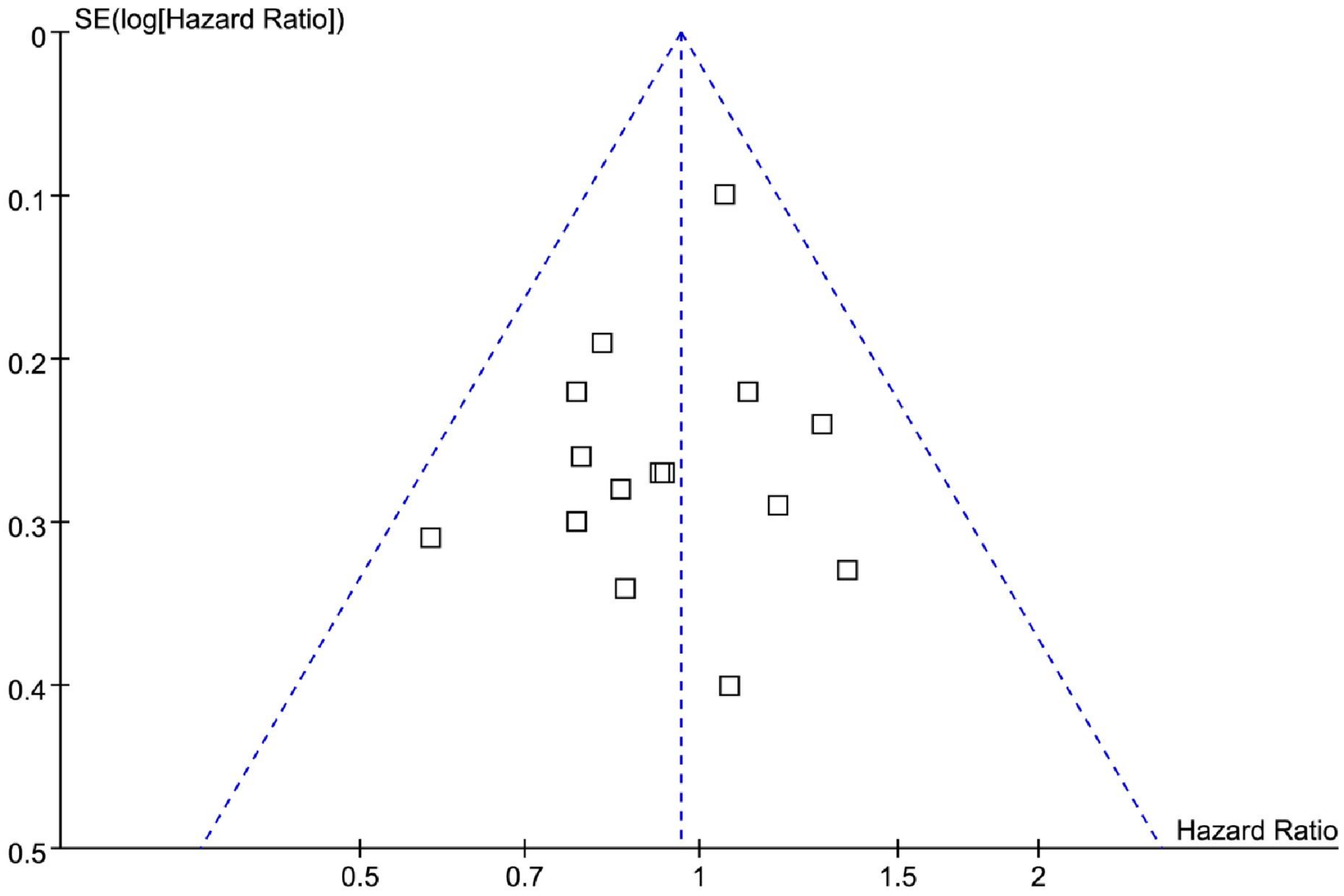

Funnel plot for all studies comparing overall survival.
Each study is marked by a dot around the overall effect estimate (vertical line). The diagonal lines represent the 95% confidence interval around the overall effect estimate. The symmetrical distribution of all dots showed that there was no significant publication bias. SE, standard error.
