# Supplementary material for: Timing of Hepatectomy for Resectable Synchronous Colorectal Liver Metastases: For Whom Simultaneous Resection Is More Suitable - A Meta-Analysis
Source: PLoS One. 2014 Aug 5;9(8):e104348. doi: 10.1371/journal.pone.0104348 (PMC4122440; doi:10.1371/journal.pone.0104348)
Supplement: Table S1 — Quality assessment of studies included in the meta-analysis. (PDF) [file pone.0104348.s010.pdf]

Table S1 Quality assessment of studies included in the meta-analysis

		Selection				Comparability		Outcome		
**Studies**	**Total score**	**Exposed cohort selection**	**Non-exposed cohort selection**	**Ascertainment of exposure**	**Outcome was not present at start of study**	**Select the most important factor**	**Controls for any additional factor**	**Assessment of outcome**	**Follow-up long enough**	**Adequacy of follow up of cohorts**
**Abbott 2012**	9	1	1	1	1	1	1	1	1	1
**Alexandrescu 2012**	7	1	1	1	1	1	1	1	0	0
**Brouquet 2010**	8	1	1	1	1	1	1	1	0	1
**Capussotti 2007**	8	1	1	1	1	1	0	1	1	1
**Chua 2004**	6	1	1	1	1	1	0	1	0	0
**de Haas 2010**	9	1	1	1	1	1	1	1	1	1
**Hu 2013**	8	1	1	1	1	1	0	1	1	1
**Luo 2010**	6	1	1	1	1	1	0	1	0	0
**Martin 2003**	7	1	1	1	1	1	1	1	0	0
**Martin 2009**	7	1	1	1	1	1	1	1	0	0
**Mayo 2013**	8	1	1	1	1	1	1	1	1	0
**Moug 2010**	7	1	1	1	1	1	1	1	0	0
**Reddy 2007**	6	1	1	1	1	1	0	1	0	0
**Slupski 2009**	6	1	1	1	1	1	0	1	0	0
**Tanaka 2004**	7	1	1	1	1	1	0	1	1	0
**Thelen 2007**	8	1	1	1	1	1	1	1	1	0
**Turrini 2007**	8	1	1	1	1	1	0	1	1	1
**Vassiliou 2007**	6	1	1	1	1	1	0	1	0	0
**Wang 2008**	8	1	1	1	1	1	1	1	1	0
**Weber 2003**	7	1	1	1	1	1	0	1	1	0
**Xu 2009**	7	1	1	1	1	1	0	1	1	0
**Yan 2007**	6	1	1	1	1	1	0	1	0	0
**Vigano 2011***	5	0	0	1	1	1	0	1	1	0
**van der Pool 2010***	4	0	0	1	1	1	0	1	0	0

Exposed cohort: patients received simultaneous resection.

Non-exposed cohort: patients received staged resection.

Ascertainment of exposure: patients' information came from reliable medical records.
Outcome was not present at start of study: patients received no colorectal or hepatic surgery before.
Select the most important factor: the study compared short-term and/or long-term outcomes between simultaneous and staged resection groups.
Controls for any additional factor: in the study, univariate and multivariate analysis was conducted to exclude the potential confounders.
Assessment of outcome: patients' information came from reliable medical records; perioperative mortality contained death in at least 30 days after the hepatectomy; if long-term survival was reported, it should be in the form of survival curves or 1, 3, 5-year rates.
Follow-up long enough: median or mean follow-up was considered long enough at least 30 months.
Adequacy of follow up of cohorts: the lost of follow up was reported, and was lower than 20%.
Quality of studies was considered high with NOS score ≥ 6. The studies with NOS score < 6 were considered low quality, and would not be included in meta-analysis.
*: studies excluded because of the low quality.
