# Supplementary material for: Timing of Hepatectomy for Resectable Synchronous Colorectal Liver Metastases: For Whom Simultaneous Resection Is More Suitable - A Meta-Analysis
Source: PLoS One. 2014 Aug 5;9(8):e104348. doi: 10.1371/journal.pone.0104348 (PMC4122440; doi:10.1371/journal.pone.0104348)
Supplement: Table S2 — Summarized prognostic factors for SCRLM within studies included. (PDF) [file pone.0104348.s011.pdf]

Table S2 Summarized prognostic factors for SCRLM within studies included

Studies	Total patients	Analysis for	Prognostic factors			
			Simultaneous resection		Delayed resection	
			Significant factors	Analysis	Significant factors	Analysis
Abbott 2012	144		-	No analysis	-	No analysis
Alexandrescu 2012	142		-	No analysis	-	No analysis
Brouquet 2010	115	OS	Liver tumor size > 3cm<br>Need for a second line of preoperative chemotherapy			Univariate
			Liver tumor size > 3cm			Multivariate
Capussotti 2007	127	OS	Male sex<br>T4<br>Multiple metastases<br>Liver metastases ≥ 4<br>Metastatic infiltration of nearby structures	Univariate	None	Univariate
			Male sex<br>T4<br>Liver metastases ≥ 4<br>Metastatic infiltration of nearby structures	Multivariate	None	Multivariate
Chua 2004	96	-	-	No analysis	-	No analysis
de Haas 2010	228	R	Liver metastases ≥ 3<br>Bilobar distribution<br>Initially unresectable metastases<br>Preop. chemotherapy<br>Timing of liver resection<br>Extrahepatic disease<br>Non-anatomical resection<br>Margin involved<br>Postop. chemotherapy			Univariate
			Liver metastases ≥ 3<br>Initially unresectable<br>Timing of liver resection			Multivariate
Hu 2013	53	-	-	No analysis	-	No analysis
Luo 2010	405	CM	None			Univariate
			None			Multivariate
Martin 2003	240	CM	Pulmonary history<br>Past abdominal surgery<br>Extent hepatic resection ≥ lobectomy<br>Timing of liver resection<br>Type of liver resection			Univariate

			Pulmonary history<br>Past abdominal surgery<br>Timing of liver resection			Multivariate
Martin 2009	230	CM	Blood transfusion<br>Extent hepatic resection ≥ lobectomy<br>Type of liver resection/ablation			Univariate
			Blood transfusion			Multivariate
Mayo 2013	976	CM	Largest CRLM >3.5 cm<br>Major hepatic resection			Univariate
			Bilateral hepatic disease<br>Major hepatic resection			Multivariate
		OS	Male sex<br>Rectal primary<br>>2 Colorectal liver metastases<br>Minor hepatic resection<br>Combined resection þ ablation			Univariate
			Male sex<br>Rectal primary<br>Minor hepatic resection<br>Combined resection þ ablation			Multivariate
Moug 2010	64	-	-	No analysis	-	No analysis
Reddy 2007	610	CM	Liver metastases ≥ 4	Univariate	-	No analysis
Slupski 2009	89	-	-	No analysis	-	No analysis
Tanaka 2004	76	OS	Resected liver volume<br>Age ≥ 70 years<br>Poorly differentiated primary/ mucinous adenocarcinoma	Univariate	Duration of operation	Univariate
			Poorly differentiated primary/ mucinous adenocarcinoma	Multivariate	None	Multivariate
Thelen 2007	219	OS	Extent hepatic resection ≥ lobectomy	Univariate	Extent hepatic resection ≥ lobectomy<br>Margin involved<br>Liver metastases ≥ 4<br>Node-positive primary	Univariate
			None	Multivariate	Node-positive primary<br>Liver metastases ≥ 4<br>Margin involved	Multivariate
Turrini 2007	119	OS	None			Univariate
Vassiliou 2007	103	-	-	No analysis	-	No analysis

Wang 2008	83	-	-	No analysis	-	No analysis
Weber 2003	97	OS	None			Univariate
Xu 2009	175	-	-	No analysis	-	No analysis
Yan 2007	103	-	-	No analysis	-	No analysis

Only statistically significant factors (P < 0.05) were listed in the table.

OS, Overall survival; CM, Complication morbidity; R, Recurrence.

No analysis, No univariate or multivariate analysis was conducted in the study.

None, univariate or multivariate was conducted in the analysis, but no statistically significant factors were found.
