# Supplementary material for: Timing of Hepatectomy for Resectable Synchronous Colorectal Liver Metastases: For Whom Simultaneous Resection Is More Suitable - A Meta-Analysis
Source: PLoS One. 2014 Aug 5;9(8):e104348. doi: 10.1371/journal.pone.0104348 (PMC4122440; doi:10.1371/journal.pone.0104348)
Supplement: Table S3 — Sensitivity analysis of postoperative mortality, morbidity and overall survival. (PDF) [file pone.0104348.s012.pdf]

Table S3 Sensitivity analysis

	Postoperative mortality		Postoperative morbidity		Overall survival	
**Studies omitted**	**Repooled RR [95%CI]**	**P**	**Repooled RR [95%CI]**	**P**	**Repooled HR [95%CI]**	**P**
**All studies included**	**1.14 [0.77, 1.69]**	**0.52**	**0.88 [0.81, 0.96]**	**0.003**	**0.96 [0.86, 1.08]**	**0.50**
**Abbott 2012**	1.10 [0.74, 1.65]	0.63	0.87 [0.80, 0.96]	0.003	-	-
**Alexandrescu 2012**	1.23 [0.81, 1.87]	0.34	0.87 [0.80, 0.95]	0.002	0.97 [0.86, 1.09]	0.60
**Brouquet 2010**	1.12 [0.75, 1.68]	0.59	0.88 [0.80, 0.96]	0.004	0.97 [0.86, 1.09]	0.63
**Capussotti 2007**	1.12 [0.75, 1.67]	0.58	0.87 [0.80, 0.95]	0.003	0.94 [0.84, 1.06]	0.34
**Chua 2004**	1.14 [0.77, 1.69]	0.52	0.89 [0.81, 0.97]	0.007	0.95 [0.85, 1.07]	0.42
**de Haas 2010**	1.14 [0.76, 1.70]	0.52	0.89 [0.82, 0.97]	0.010	0.95 [0.85, 1.07]	0.40
**Hu 2013**	1.14 [0.77, 1.69]	0.52	0.88 [0.81, 0.96]	0.006	0.96 [0.86, 1.08]	0.48
**Luo 2010**	1.18 [0.78, 1.78]	0.42	0.88 [0.80, 0.97]	0.007	-	-
**Martin 2003**	1.17 [0.78, 1.76]	0.45	0.90 [0.82, 0.99]	0.020	-	-
**Martin 2009**	1.15 [0.77, 1.71]	0.50	0.87 [0.79, 0.95]	0.002	-	-
**Mayo 2013**	1.30 [0.81, 2.06]	0.27	0.86 [0.79, 0.94]	0.001	0.92 [0.80, 1.06]	0.24
**Moug 2010**	1.14 [0.77, 1.69]	0.52	0.89 [0.81, 0.97]	0.007	-	-
**Reddy 2007**	1.04 [0.68, 1.59]	0.84	0.87 [0.80, 0.96]	0.003	-	-
**Slupski 2009**	1.15 [0.77, 1.72]	0.50	0.88 [0.80, 0.96]	0.003	0.96 [0.86, 1.08]	0.54
**Tanaka 2004**	1.14 [0.77, 1.69]	0.52	0.87 [0.80, 0.95]	0.002	0.98 [0.87, 1.10]	0.72
**Thelen 2007**	1.00 [0.65, 1.52]	0.99	0.88 [0.81, 0.96]	0.002	0.95 [0.85, 1.07]	0.41
**Turrini 2007**	1.17 [0.78, 1.76]	0.45	0.88 [0.81, 0.96]	0.005	0.98 [0.87, 1.10]	0.71
**Vassiliou 2007**	1.14 [0.77, 1.69]	0.52	0.87 [0.80, 0.96]	0.003	-	-
**Wang 2008**	1.14 [0.77, 1.69]	0.52	0.87 [0.80, 0.95]	0.002	0.96 [0.86, 1.08]	0.53
**Weber 2003**	1.14 [0.77, 1.69]	0.52	0.88 [0.81, 0.96]	0.004	0.96 [0.86, 1.08]	0.52
**Xu 2009**	1.14 [0.76, 1.73]	0.53	0.86 [0.79, 0.94]	0.001	0.98 [0.87, 1.10]	0.69
**Yan 2007**	1.14 [0.77, 1.69]	0.52	0.88 [0.81, 0.96]	0.005	0.97 [0.86, 1.09]	0.57

Sensitivity analyses were performed by consecutively omitting every study from all the others in the meta-analysis (leave-one-out procedure).
