# Supplementary material for: Timing of Hepatectomy for Resectable Synchronous Colorectal Liver Metastases: For Whom Simultaneous Resection Is More Suitable - A Meta-Analysis
Source: PLoS One. 2014 Aug 5;9(8):e104348. doi: 10.1371/journal.pone.0104348 (PMC4122440; doi:10.1371/journal.pone.0104348)
Supplement: Information S1 — Search strategy for “PubMed”, “Web of Science” and “Embase”. (PDF) [file pone.0104348.s014.pdf]

Information S1

Search strategies

Search strategy for “PubMed” as follows:
(((colorectal neoplasms[MeSH Terms]) AND metastas*[Title/Abstract]) AND (simultaneous OR synchronous[Title/Abstract])) AND (resection* OR hepatectomy[Title/Abstract]) AND (("2000/01/01"[PDat] : "2013/04/31"[PDat]))

Search strategy for “Web of Science” as follows:
Topic=(colorectal cancer) AND Topic=(metastas*) AND Topic=(simultaneous OR synchronous) AND Topic=(resection OR hepatectomy)
Timespan=2000-2013. Databases=SCI-EXPANDED, SSCI, A&HCI, CPCI-S, CPCI-SSH, CCR-EXPANDED, IC.

Search strategy for “Embase” as follows:
(colorectal cancer and metastas*).ti. and (simultaneous or synchronous).ab. and (resection or hepatectomy).ab.
