# Supplementary material for: Timing of Hepatectomy for Resectable Synchronous Colorectal Liver Metastases: For Whom Simultaneous Resection Is More Suitable - A Meta-Analysis
Source: PLoS One. 2014 Aug 5;9(8):e104348. doi: 10.1371/journal.pone.0104348 (PMC4122440; doi:10.1371/journal.pone.0104348)
Supplement: Information S2 — Correction of baseline imbalance in terms of number of liver metastases. (PDF) [file pone.0104348.s015.pdf]

Information S2

Correction of baseline imbalance in terms of number of liver metastases

We conducted this analysis to correct the baseline imbalance on number of liver metastases by respectively calculating the postoperative morbidity of patients with ≤ 3 or > 3 metastases after simultaneous or staged resection.

Take the patients with ≤ 3 metastases in simultaneous group for example. 6 studies reported data of number of liver metastases as "≤ 3 vs. > 3". The data were sorted out in format of the proportion of patients with ≤ 3 metastases and the corresponding morbidity (Details in Table SS1). Considering that the postoperative morbidity was significantly related with the number of liver metastases, the proportion of patients with ≤ 3 metastases should have linear correlation with the corresponding morbidity (Details in Figure SS1A). Each study was considered as an observed value, and a linear regression model was constructed with 95% confidence interval (Linear equation in Table SS2). In this liner equation, independent variable was the proportion of patients with ≤ 3 metastases, and the dependent variable was postoperative morbidity. Each study was given the same weight in the model because all studies included in the meta-analysis were in high quality, with a sample size over 50. Point estimate for the independent variable = 1, we got the postoperative morbidity of patients with ≤ 3 metastases after simultaneous resection (17.2%, 95%CI = [0 - 37.3%]). And point estimate for the independent variable = 0, we got the morbidity of patients with > 3 metastases (49.4%, 95%CI = [9.4% - 89.5%]).

The same method was used to estimate the morbidity of patients with ≤ 3 metastases (13.8%, 95%CI = [0 - 28.6%]) or > 3 metastases (50.8%, 95%CI = [33.8% - 67.9%]) after staged resection (Linear equation in Table SS3).

We also conducted analyses of studies reported number of liver metastases as "Single vs. Multiple". The results showed no significant correlation in simultaneous group (P = 0.250) or staged group (P = 0.193), which meant that "Single vs. Multiple" was not a good cut-off value for number of liver metastases.

Table SS1 Data sorted out as the proportion of patients with ≤ 3 metastases, and the corresponding morbidity

	Simultaneous resection group				Staged resection group			
Studies	No. of patients with ≤ 3 liver metastases	Total No. of patients	Proportion of patients with ≤ 3 metastases	Postoperative morbidity	No. of patients with ≤ 3 liver metastases	Total No. of patients	Proportion of patients with ≤ 3 metastases	Postoperative morbidity
Thelen 2007	34	40	0.85	0.18	45	179	0.68	0.25
Turrini 2007	42	57	0.74	0.21	19	62	0.44	0.31
Wang 2008	32	37	0.86	0.24	9	46	0.76	0.20
Weber 2003	29	35	0.82	0.23	20	62	0.63	0.32
Xu 2009	68	96	0.71	0.34	22	79	0.67	0.28
Yan 2007	37	73	0.51	0.31	13	30	0.23	0.43

Table SS2 Linear equation of proportion of patients with ≤ 3 metastases and the corresponding morbidity in simultaneous resection group

Coefficients[a]

Model	Unstandardized Coefficients		Standardized Coefficients	t	Sig.	95.0% Confidence Interval for B		Correlations			Collinearity Statistics	
	B	Std. Error	Beta			Lower Bound	Upper Bound	Zero-order	Partial	Part	Tolerance	VIF
1 (Constant)	.494	.134		3.689	.021	.122	.866					
≤ 3 metastases	-.323	.176	-.675	-1.827	.142	-.813	.167	-.675	-.675	-.675	1.000	1.000

a. Dependent Variable: Morbidity

Table SS3 Linear equation of proportion of patients with ≤ 3 metastases and the corresponding morbidity in staged resection group

Coefficients[a]

Model		Unstandardized Coefficients		Standardized Coefficients	t	Sig.	95.0% Confidence Interval for B		Correlations			Collinearity Statistics	
		B	Std. Error	Beta			Lower Bound	Upper Bound	Zero-order	Partial	Part	Tolerance	VIF
1	(Constant)	.509	.049		10.298	.001	.372	.646					
	≤ 3 metastases	-.371	.083	-.913	-4.476	.011	-.601	-.141	-.913	-.913	-.913	1.000	1.000

a. Dependent Variable: Morbidity

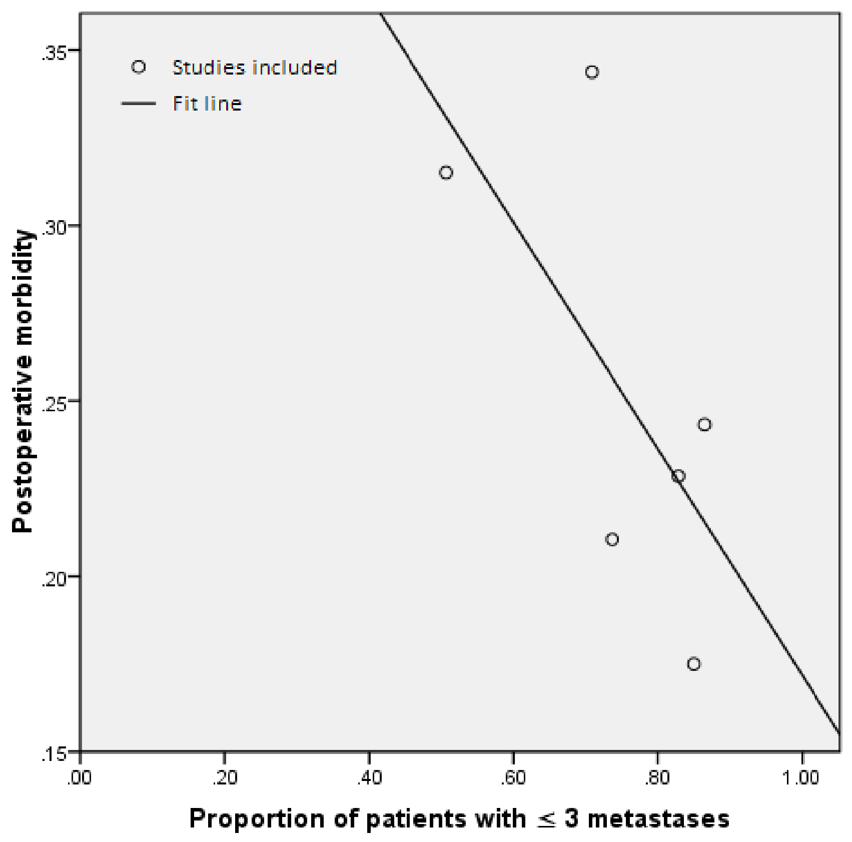

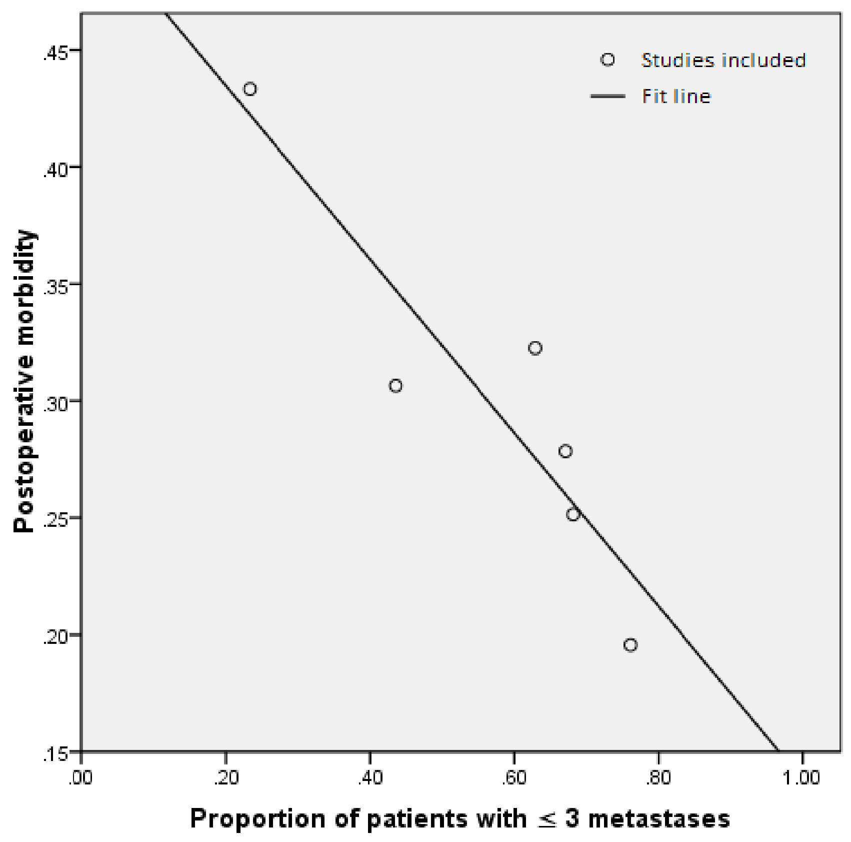

Figure SS1A Linear correlation between proportion of patients with ≤ 3 metastases and the postoperative morbidity in simultaneous resection group.	Figure SS1B Linear correlation between proportion of patients with ≤ 3 metastases and the postoperative morbidity in staged resection group.
